# Investigations into Roman Mortar from Sucidava, Romania

**DOI:** 10.3390/ma19194168

**Published:** 2026-09-29

**Authors:** Flavio Nicoale Finta, Marioara Abrudeanu, Denis Aurelian Negrea, Sorin Georgian Moga, Isabela Victoria Maris, Florentina Popa, Ecaterina Magdalena Modan, Dorin Grecu, Ionela Cristina Gheroghe, Marius Lungescu, Mircea Negru, Mircea Ionut Petrescu

**Affiliations:** 1 Doctoral School of Materials Science and Engineering, National University of Science and Technology POLITEHNICA Bucharest, Splaiul Independenței, No. 313, 060042 Bucharest, Romania; nicolae.finta@stud.sim.upb.ro (F.N.F.); abrudeanu@gmail.com (M.A.); ionela.gheorghe1709@stud.sim.upb.ro (I.C.G.); marius.lungescu@stud.sim.upb.ro (M.L.); 2 Regional Center of Research & Development for Materials, Processes and Innovative Products Dedicated to the Automotive Industry (CRC&D-AUTO), Pitești University Centre, National University of Science and Technology POLITEHNICA Bucharest, 110040 Pitești, Romania; sorin_georgian.moga@upb.ro (S.G.M.); florentina.popa1702@upb.ro (F.P.); ecaterina.modan@upb.ro (E.M.M.); 3 Department of Mineralogy, Faculty of Geology and Geophysics, University of Bucharest, 1 N. Bălcescu Ave., 011401 Bucharest, Romania; izabela@contentlogic.ro; 4 Faculty of Theology, Letters, History and Arts, National University of Science and Technology POLITEHNICA Bucharest, Splaiul Independenței, No. 313, 060042 Bucharest, Romania; dorin.grecu@upb.ro; 5 Department of Law, Political and Administrative Sciences, Spiru Haret University Bucharest, Soseaua Berceni, No. 34, 041914 Bucharest, Romania; mircea_negru_arch@yahoo.com; 6 Department of Engineering and Management of Metallic Materials Casting, Faculty of Materials Science and Engineering, National University of Science and Technology POLITEHNICA Bucharest, Splaiul Independenței, No. 313, 060042 Bucharest, Romania; ionut.petrescu@upb.ro

**Keywords:** Roman mortar, Sucidava, *opus caementicium*, *opus signinum*, composition, microstructure

## Abstract

This study presents an interdisciplinary investigation of mortars from the Roman fortress of Sucidava, constructed in the 3rd century AD along the Danube River, at the northern frontier of the Roman Empire. The mortars were characterized using a multi-analytical approach, including macroscopic examination, optical microscopy, cathodoluminescence microscopy, scanning electron microscopy coupled with energy-dispersive X-ray spectroscopy (SEM-EDS), and qualitative phase analysis by X-ray diffraction (XRD). The results highlight the presence of two distinct mortar types: (i) a gray, coarse-grained masonry mortar consistent with *opus caementicium*, covered by a finishing layer, and (ii) a fine-grained red plaster mortar analogous to *opus signinum*, as described in ancient architectural treatises. The structural and compositional features of mortars sampled from the eastern wall of the Sucidava Fortress confirm the persistence of advanced Roman mortar technology in this northern frontier region, even after the division of the Roman Empire. The substitution of volcanic ash with bone ash led to the incorporation of phosphorus-bearing compounds, which, according to recent studies, contribute to enhanced mortar performance.

## 1. Introduction

Roman mortar was first used during the early Roman Republic (2nd century BC) for masonry and stone constructions. Its initial composition was relatively simple, consisting of slaked lime, sand, and water, and it exhibited relatively low mechanical strength, particularly in humid environments. Over time, the Romans continuously improved mortar technology, drawing inspiration from Greek and Etruscan practices.

A major advancement occurred during the 2nd–1st centuries BC, when the Romans introduced pozzolana (volcanic ash from the Pozzuoli area near Naples), marking the emergence of hydraulic mortar capable of hardening under water. This innovation enabled the construction of large-scale structures, including harbors and aqueducts [1,2,3,4,5,6,7].

The peak of Roman mortar technological development occurred between the 1st and 3rd centuries AD, coinciding with the height of the Roman Empire. During this period, the Romans commonly used mixtures consisting of one part lime and two to three parts pozzolana from the Pozzuoli region (Gulf of Naples), combined with aggregates such as gravel, crushed bricks, and ceramic fragments, resulting in mortar commonly referred to as *opus signinum*.

The works of Vitruvius and Pliny the Elder describe several types of *opus*: *opus caementicium,* Roman concrete (lime + pozzolana + aggregates); *opus signinum,* a fine reddish plaster or flooring material (containing brick dust); and *opus reticulatum* and *opus latericium,* used for decorative masonry with mortar [1,2]. Vitruvius also described hydraulic concrete (*opus caementicium*) used in marine structures (harbors, breakwaters, and underwater foundations), emphasizing its resistance to seawater and the essential role of volcanic ash (*pulvis puteolanus*), which, in reaction with lime, imparts hydraulic properties [1].

Classical authors such as Strabo, Pliny the Elder, and Seneca documented the use of volcanic materials and highlighted the durability of Roman constructions. The addition of ceramic fragments and pozzolanic materials significantly enhanced mechanical strength and durability. Knowledge regarding optimal raw materials and mix proportions for hydraulic concrete spread throughout the Mediterranean basin through the mobility of Roman engineers, the circulation of technical manuals, and, most importantly, through the transmission of construction practices and technological traditions to occupied and neighboring territories, as well as to later historical periods [3,4,5,6,7,8,9,10,11,12,13,14,15,16].

Subsequently, the decline in the quality of Roman mortar is closely associated with the division of the Roman Empire. Political and economic fragmentation led to the loss of centralized control, disruption of trade networks, and the gradual disappearance of standardized technical practices. This degradation was more pronounced in the Western Roman Empire than in the Eastern Empire. After the 4th century AD, reduced access to high-quality pozzolanic materials, weakening administrative structures, and economic instability contributed significantly to the decline in mortar performance.

Whereas the unified Empire had supported an integrated market ensuring the supply and long-distance distribution of high-quality pozzolanic ash from central Italy, along with suitable lime and aggregates, the economic crisis of the 4th–5th centuries AD led to the collapse of long-distance trade, particularly in the Western Roman Empire. As a consequence, access to high-quality materials diminished. Construction activity declined overall, with fewer large-scale infrastructures such as harbors, baths, and aqueducts being built, and some settlements were even abandoned. A preference emerged for faster-setting, lower-quality mortars to meet immediate construction needs [17,18,19,20].

In contrast, in the Eastern Roman (Byzantine) Empire, administrative continuity and a relatively stable economy allowed the persistence of Roman technical traditions, resulting in mortars that were generally superior to their Western counterparts. Following the fall of Rome, the knowledge of hydraulic mortar production was gradually lost in Western Europe, and medieval builders reverted to simple air-lime mortars. Hydraulic mortar technology was rediscovered only in the 18th century, notably by John Smeaton in 1756 during the construction of the Eddystone Lighthouse, when he re-established the pozzolanic principle. In the 19th century, Joseph Aspdin patented Portland cement, representing a direct technological evolution from Roman mortar principles.

Further developments led to the widespread use of Portland cement-based mortars and lime–cement mortars, particularly during the 20th century. While these materials provide superior mechanical strength, they may exhibit increased stiffness and reduced compatibility with historic masonry. Modern design standards emphasize the importance of appropriate mix design and long-term durability. For many decades, Portland cement mortars were considered superior to Roman mortars; however, recent research has increasingly challenged this assumption.

Recent research has demonstrated that many properties of modern mortars are not necessarily superior to those of Roman mortar. Roman mortar can be considered a “living” material, undergoing slow and continuous chemical reactions over time, whereas Portland cement mortar is an industrial material characterized by rapid reactions [12]. Roman mortar typically exhibits moderate compressive strength, high elasticity, and an enhanced ability to accommodate structural movements [16].

Under humid or marine conditions, Roman mortar demonstrates exceptional durability over millennial timescales, whereas Portland cement mortar generally exhibits a lifespan on the order of decades to centuries, being more susceptible to degradation from sulfates, salts, and especially temperature variations associated with freeze–thaw cycles. From an environmental perspective, Roman mortar is significantly more sustainable due to lower calcination temperatures for lime production, reduced CO_2_ emissions, and the use of locally sourced materials. In contrast, Portland cement has a substantial environmental impact, owing to energy-intensive manufacturing processes and high associated CO_2_ emissions.

Recent studies have provided insight into the durability of Roman mortar and highlight the importance of its scientific reassessment. Research has shown that pozzolanic reactions within the mortar, calcium hydroxide (derived from lime), and aggregate components lead to the formation of mineral phases that strengthen the structure and enhance durability. Specifically, the interaction between calcium hydroxide, silica, and water results in the formation of calcium silicate hydrate (C–S–H) gel, while the presence of alumina (Al_2_O_3_) promotes the formation of calcium–aluminosilicate hydrate (C–A–S–H) phases. These hydrated compounds are primarily responsible for the mechanical strength and long-term durability of mortars.

xCa(OH)_2_ + yAl_2_O_3_ · zSiO_2_ + (n − x)H_2_O = (CaO)_x_ · (SiO_2_)z · (Al_2_O_3_)y (H_2_O)_n−x_ (or C–A–S–H gel) (1)

The binding phases formed, characterized by a disordered, poorly crystalline structure, namely C–S–H and C–A–S–H, contribute to the long-term increase in mechanical strength, chemical stability, and durability [8,9,10,11,12,13,14,15]. The presence of siliceous aggregates has also been confirmed by other studies [16,17,18,19].

The present interdisciplinary study of mortar from Sucidava aims, through advanced characterization techniques, to classify the material within established Roman mortar typologies and to evaluate its properties in relation to those of mortars produced in Italy during the peak period of hydraulic mortar technology.

**Figure 1:**
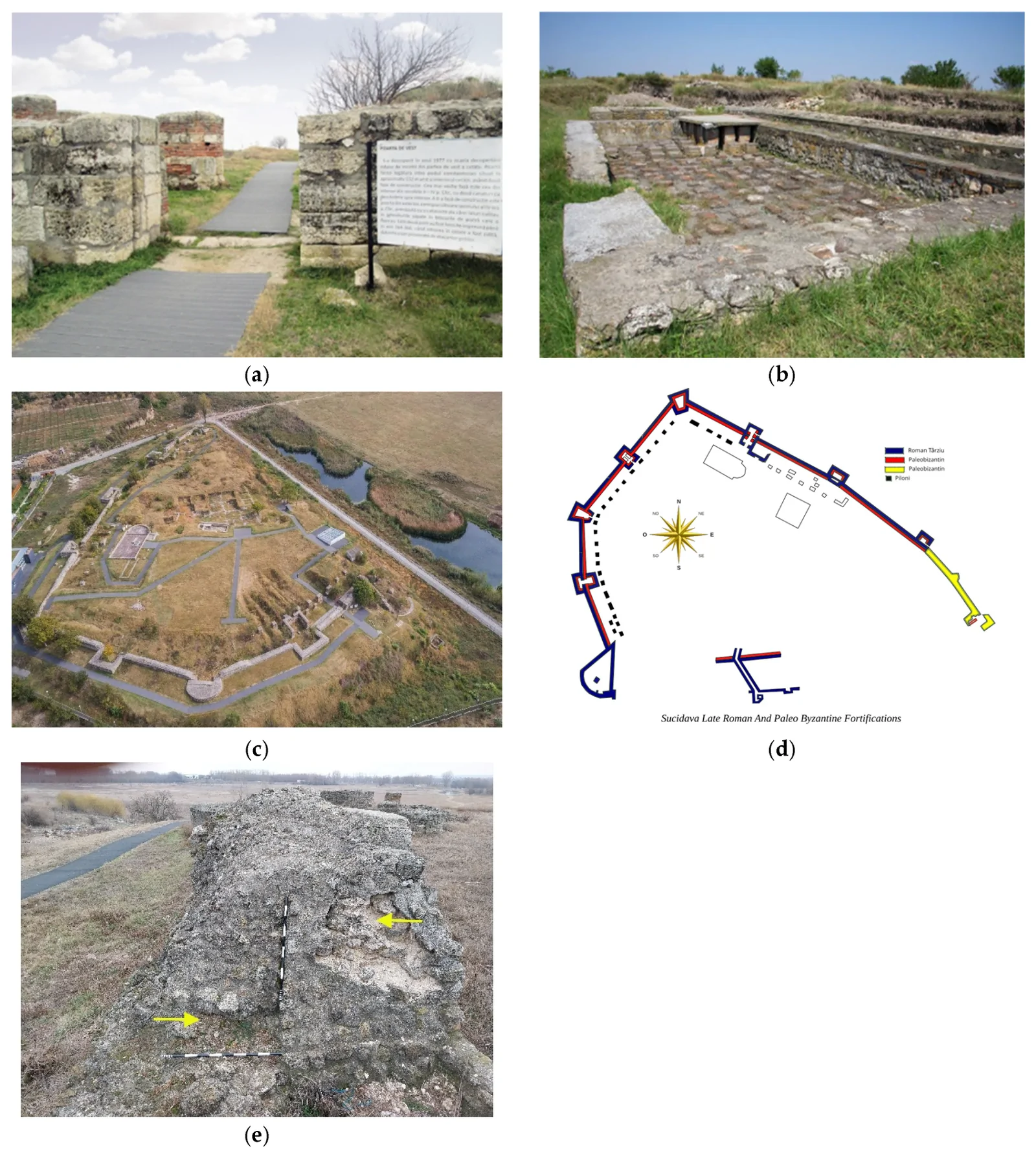
Sucidava Fortress: (**a**,**b**)—interior views of the fortress; (**c**) aerial view; (**d**) plan of the fortress [https://ro.wikipedia.org/wiki/Sucidava_(Corabia), accessed on 2 September 2026]; (**e**) exterior wall (marked by the yellow arrow on the left) and interior wall (marked by the yellow arrow on the right) [archive Negru M photo].

The Sucidava Fortress (*Sykibida*, *Σucidava*) (Figure 1) was built on an ancient settlement site inhabited since the Neolithic period, as evidenced by artifacts belonging to the Vădastra culture (4th millennium BC) and the Celei cultural group (second half of the 3rd millennium BC). The fortress, located today in the Celei district of Corabia, lies on the northern bank of the Danube River, which represented the northern *limes* (frontier) of the Roman Empire [20,21,22].

During the reign of Emperor Aurelian (270–275 AD), the first defensive fortification at Sucidava was constructed on the site of a Roman necropolis (dated to 86 AD), associated with the Legio V Alaudae, which had been destroyed during conflicts with the Dacians under King Decebalus. The fortress was later restored under Emperor Constantine the Great (306–337 AD), who also commissioned the construction of a stone bridge across the Danube.

Inaugurated in the summer of 328 AD, the bridge had a length of approximately 2437.5 m and connected the northern and southern banks of the Danube [23]. In this region of the Roman Empire, the fortress played a defensive and strategic role, overseeing and controlling river crossings while ensuring the connection between the provinces of Dacia and Moesia, located south of the Danube.

Following the Roman withdrawal from Dacia in the 3rd century AD, Sucidava continued to function, particularly during the Byzantine period (5th–6th centuries AD), serving as a bridgehead north of the Danube. The fortification controlled river crossings for several centuries and played a significant role in the Romanization of Dacia.

In 1981, on the E–F curtain, at about 3.00 m west of the inner tower, the outer defensive wall, which is later, overlapped the inner defensive wall, stratigraphically indicating that it is later (note 100). The archaeological research carried out in this sector, and the stratigraphic context indicated above, indicates that the inner wall belongs to the first phase of the Roman-Byzantine fortress, as framed by archaeologists. The outer wall belongs to the second phase of the fortification, respectively the centuries (Toropu and Tătulea 1987 [22], pp. 83–84).

According to the results of archaeological research from 1966–1985, based on archaeological materials and discovered coins, the first phase of the Roman-Byzantine fortress corresponding to the inner wall was chronologically dated to the years 265/274–323, and the later outer wall that overlaps the first one belongs to a reconstruction of the fortress enclosure, which was chronologically dated to the years 324/328–378/382 [22].

## 2. Materials and Methods

**Figure 2:**
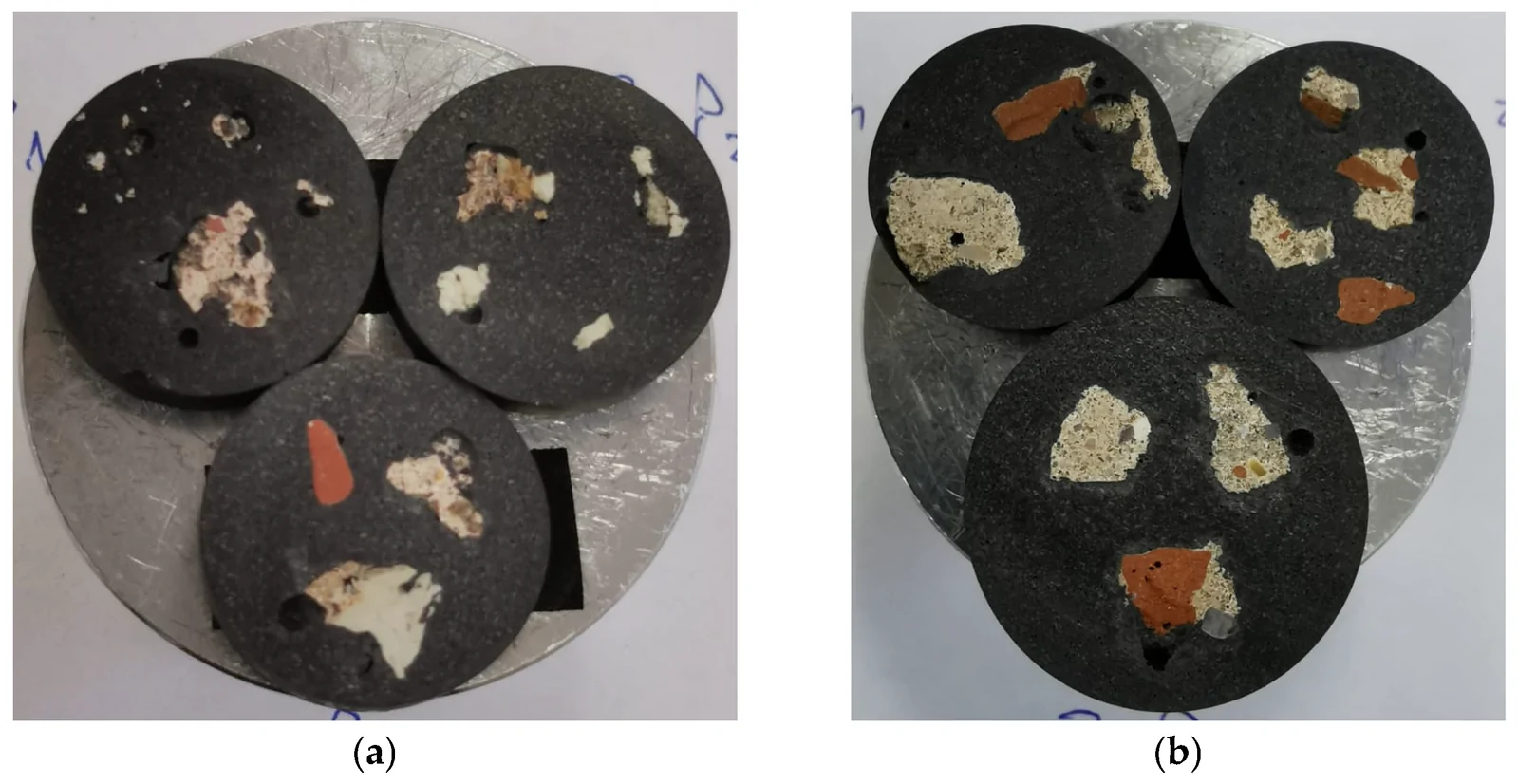
Mortar samples collected from Sucidava, embedded in conductive resin for microscopy analysis: (**a**) first batch subjected to SEM-EDS, (**b**) second batch subjected to SEM-EDS.

The materials used in this experimental study consist of mortar samples collected from the eastern wall of Sucidava Fortress, preserved from the Roman period (Figure 2). Sampling was conducted in full compliance with legislation regarding the protection of historical monuments [23,24]. The characterization of the mortars was performed through macroscopic examination, optical microscopy for grain size determination, cathodoluminescence (CL) microscopy [25,26,27], scanning electron microscopy with energy-dispersive spectroscopy (SEM-EDS) [28,29,30,31,32,33,34,35,36,37,38], and qualitative phase analysis by X-ray diffraction (XRD) [39,40,41,42,43,44].

Optical microscopy (OM) characterization was carried out under polarized light using a Zeiss Observer A1m optical microscope (Carl Zeiss Microscopy GmbH, Jena, Germany), allowing magnifications of up to 1000×, observation in bright field, dark field, and polarized light, as well as image acquisition using a Canon camera (Canon Inc., Tokyo, Japan).

Cathodoluminescence microscopy (CL) was performed using a Nikon E400 optical microscope (Nikon, Tokyo, Japan), equipped with a cold cathode cathodoluminescence device (CL 8200 MK 3A, Cambridge Image Technology Ltd., Cambridge, UK). Images were captured with a COOLPIX 950 (Nikon Corporation, Tokyo, Japan) digital photomicrograph system. The interpretation of cathodoluminescence results is qualitative.

The characterization of mortar by scanning electron microscopy coupled with energy-dispersive spectroscopy (SEM-EDS) was performed using a HITACHI SU5000 scanning electron microscope, equipped with a backscattered electron detector and an EDS module for elemental analysis (Hitachi Group, Tokyo, Japan). SEM-EDS data were obtained using a 25 kV accelerating voltage, 128 μA emission current and a working distance of approx. 10 mm. The SEM micrographs have been obtained at different working distances. The samples were analyzed in variable pressure mode at 30 Pa, without sputtering, using a backscattered electron detector for SEM micrographs, with a resolution of 3 nm obtained at 15 kV in variable pressure mode, measured on gold particles on carbon, together with an SDD energy dispersive X-ray detector (EDS) with 50 mm^2^ active surface area and a resolution of 125 eV measured on Mn Kα line. EDS analysis was performed using Oxford AZtec software, version 3.1 (Oxford Instruments NanoAnalysis, High Wycombe, UK). Chemical elemental concentrations are presented in wt%.

X-ray diffraction (XRD) analysis was employed to identify the crystalline phases present in the mortar components and, where applicable, the paint layer. XRD measurements were conducted using an XPERT PRO MPD 3060 system (Panalytical, Almelo, The Netherlands), equipped with a Cu X-ray tube (Kα = 0.154051 nm). The step size was set to 0.05° over a 2θ scanning range of 15–85°. The measurement configuration was as follows: the X-ray generator was operated at 45 kV and 40 mA, utilizing a vertical goniometer, a standard sample holder without rotation, an incident slit of 1 mm, and receiving and scattering slits set to the open position to maximize intensity. Soller slits of 2.5° were used to limit axial divergence, along with a D/teX Ultra 1D detector equipped with a monochromator. Background subtraction was performed using the fitting method, and peak identification was carried out using the second derivative method (sigma cut 3.0). A single scan was performed for each sample. Phase identification, of a qualitative nature, was performed using the PDXL2 software, version 2.9.2.0 (Rigaku Corporation, Tokyo, Japan) in conjunction with the PDF4+ 2022 database (International Centre for Diffraction Data).

## 3. Experimental Results and Discussion

The mortar samples collected from Sucidava (Table 1) include gray masonry mortars, reddish (brick-colored) mortars, and gray mortars with a tightly adherent reddish layer (Figure 2).

**Figure 3:**
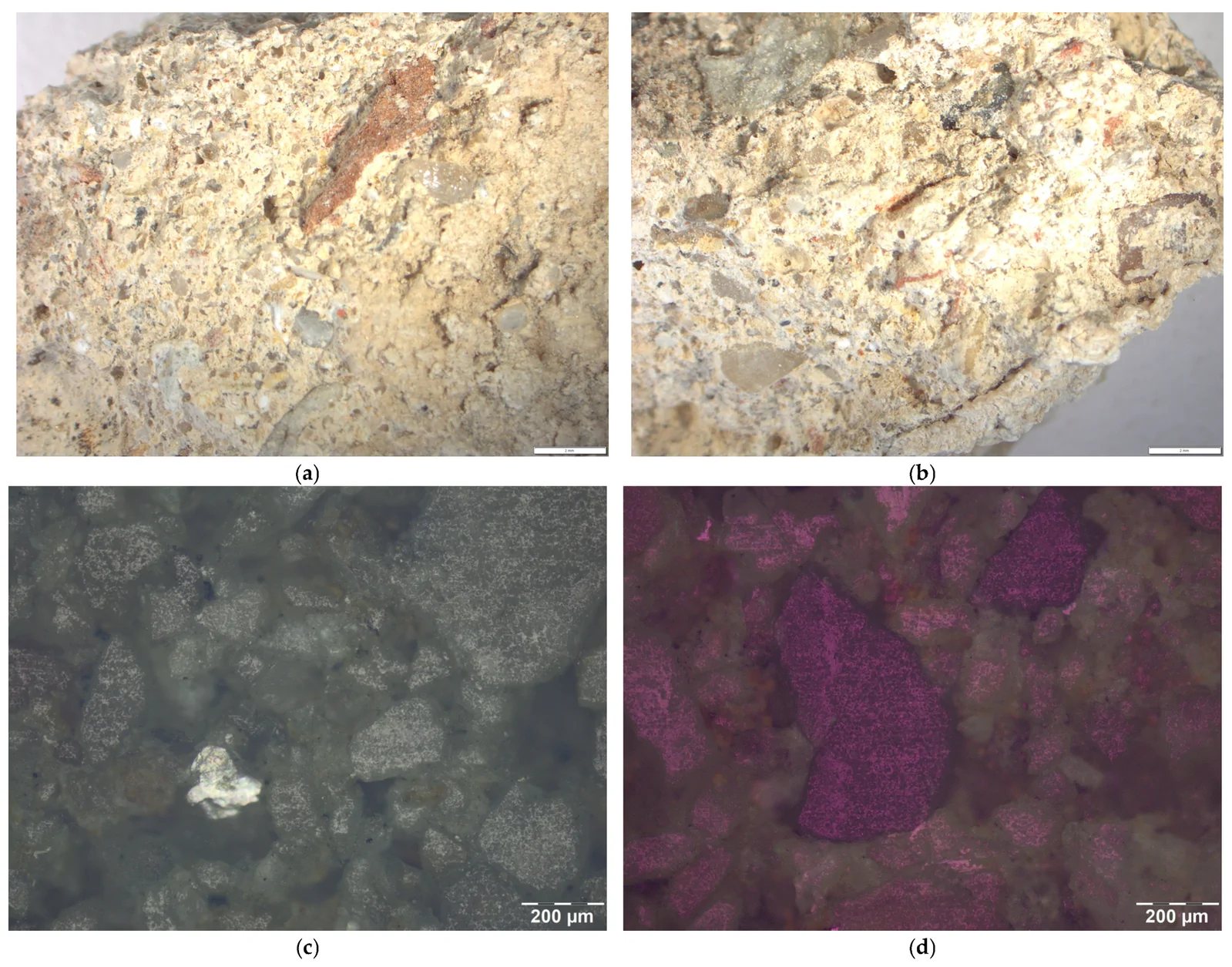
Masonry mortar samples collected from Sucidava: (**a**,**b**) macroscopic structure; (**c**,**d**) microstructure under polarized light.

The masonry mortar contains large aggregates of gray and red–brick color (suggesting stone and brick fragments) within a matrix of much finer grains. The granulometric analysis (Figure 3) highlights a high concentration of fine to medium sand-sized grains, with angular to subangular shapes, moderately sorted, and the presence of larger aggregates.

Characterization of the masonry mortar samples revealed the presence of two distinct material types: the coarse masonry mortar and a finer mortar layer functioning as a finishing plaster (Figure 3a).

Optical microscopy (OM) analysis of the masonry mortar (Figure 3) highlights particles of various shapes and sizes embedded within the mortar matrix, including the presence of bright white particles.

**Figure 4:**
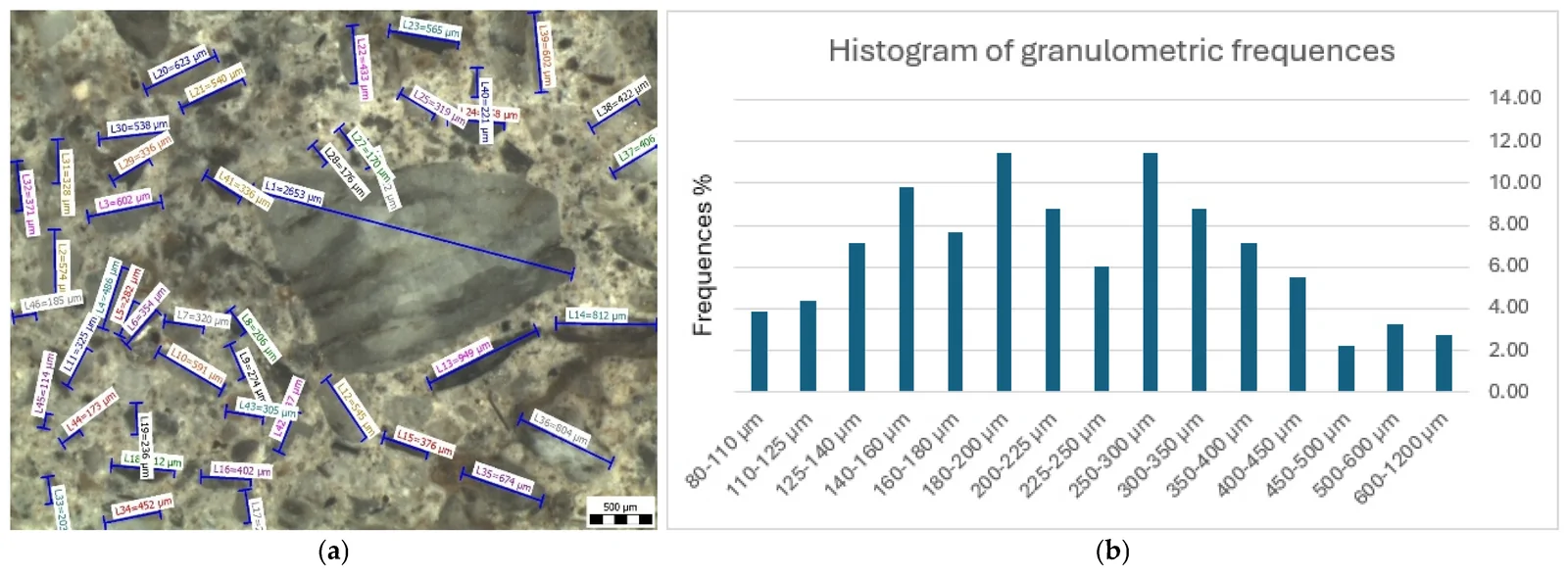
(**a**) Optical microscopy analysis and (**b**) particle size distribution of the masonry mortar (sample P13), based on optical microscopy analysis, showing a predominance of fine to medium sand-sized grains and the presence of larger aggregates.

The granulometric analysis was performed by direct measurements on the polished sample, using the Olympus BX51M optical microscope (Olympus Corp., Tokyo, Japan), equipped with the Quick Photo 3.2 measurement software (Figure 4). The measurements were carried out by Maris I., within the Sedimentology Laboratory of the Faculty of Geology and Geophysics, in Bucharest, Roamania. The granulometric values vary between 80 and 1110 µm, which corresponds to the granulometric classes from fine to coarse sand, with an average of 259.9 µm, specific to medium sand. The standard deviation value defines the degree of sorting of the material involved. The value of 145.4 µm (=2.78 phi units) expresses a very poor sorting of the sedimentary material used in the mortar preparation.

**Figure 5:**
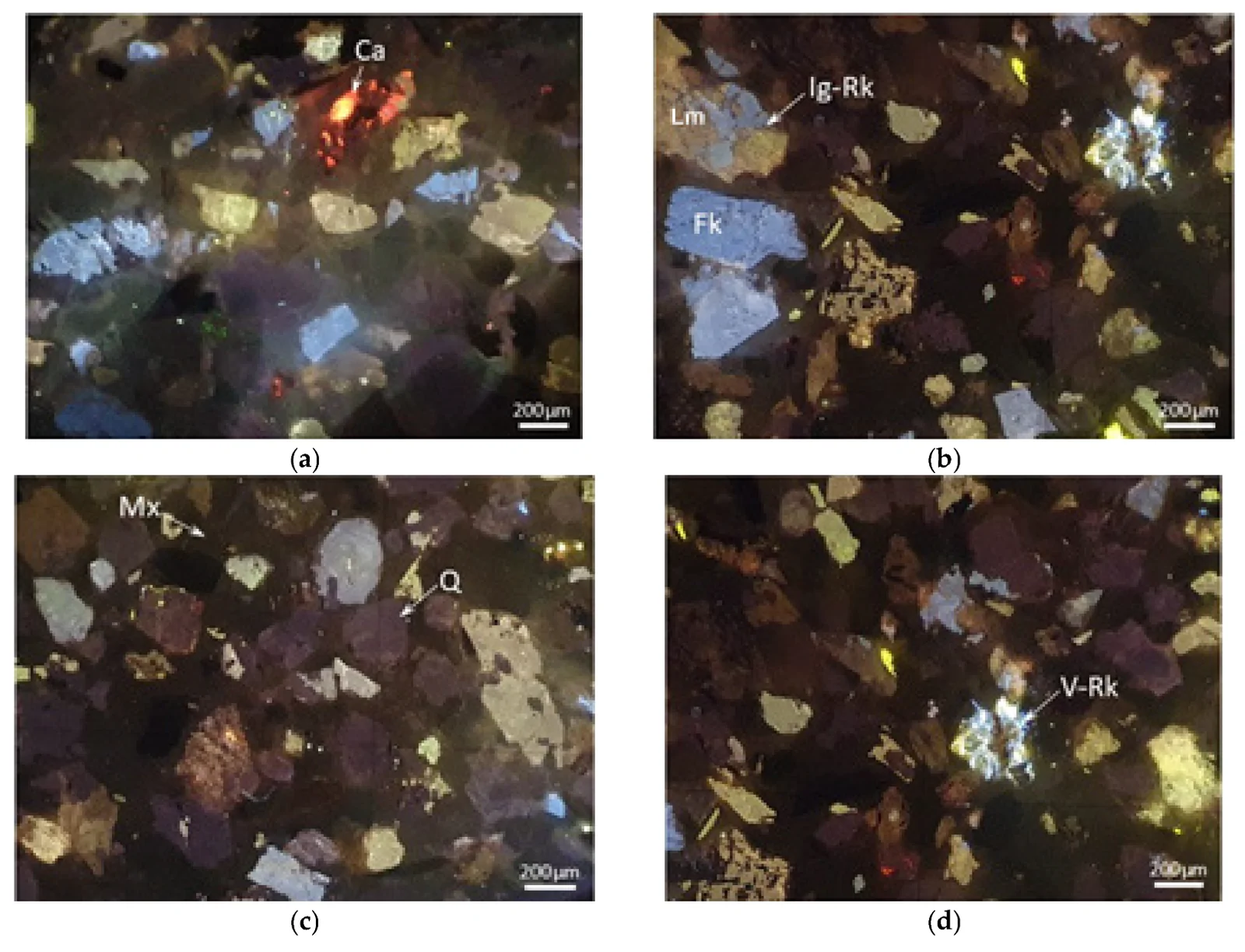
Cathodoluminescence analysis of the mortar. (**a**) Ca—calcite (**b**) Lm—metamorphic lithoclasts, Fk—alkali feldspar, Ig-Rk—igneous rock fragments (**c**) Mx—matrix, Q—quartz grains (**d**) V-Rk—volcanic rock fragments with vesicles.

Mineralogical characterization by cathodoluminescence (CL) microscopy highlights a high concentration of fine to medium sand-sized grains with angular to sub-angular shapes, moderately sorted (Figure 5). The mineralogical composition of the mortar is dominated by quartz, with subordinate feldspars, metamorphic lithoclasts, and other minerals: Fk—alkali feldspar (K, Na, Al, Si, O), Fp—plagioclase feldspar (Na, Ca, Al, Si, O), Ca—calcite, C—carbonized plant fragments, Mx—matrix, and Lm—metamorphic lithoclasts (Figure 5).

Some minerals undergo partial alteration or thermal transformation, with Si and Al becoming partially amorphous and reactive. These reactive phases interact with lime, giving the mortar its pozzolanic behavior. Consequently, andesitic rocks or tuffs rich in volcanic glass can serve as excellent sources of pozzolanic material.

**Figure 6:**
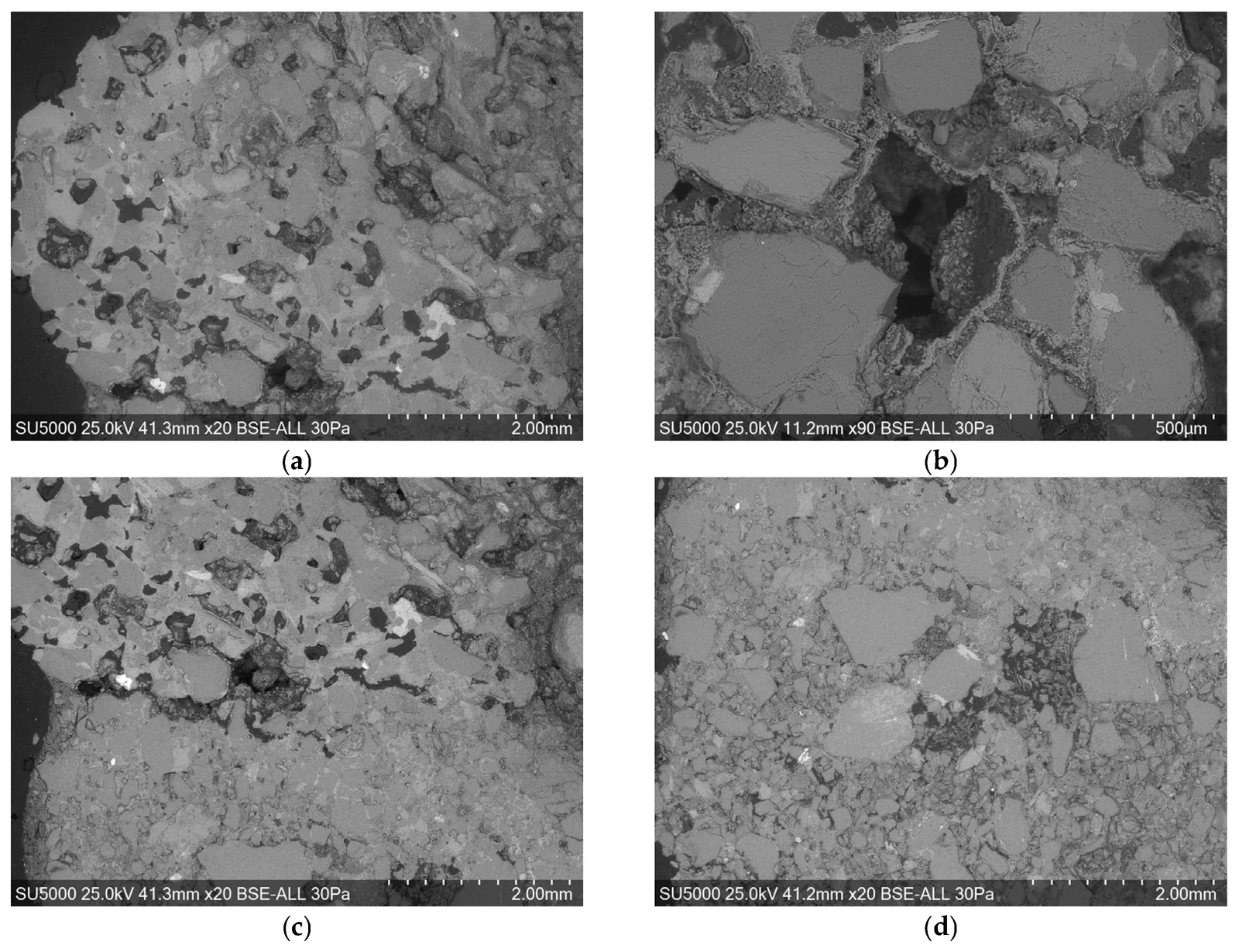
SEM morphology of mortar with plaster layer: (**a**) plaster layer; (**b**) microstructure of the plaster layer; (**c**) mortar–plaster interface; (**d**) detailed microstructure.

Morphological characterization by SEM of the masonry mortar samples revealed the microstructure of both the coarse masonry mortar and the finer exterior plaster layer, used as a protective coating (Figure 6). Sand particles in the plaster layer are less abundant, finer, and well bonded within the lime matrix, contributing to the compactness and protective function of the finishing layer.

**Figure 7:**
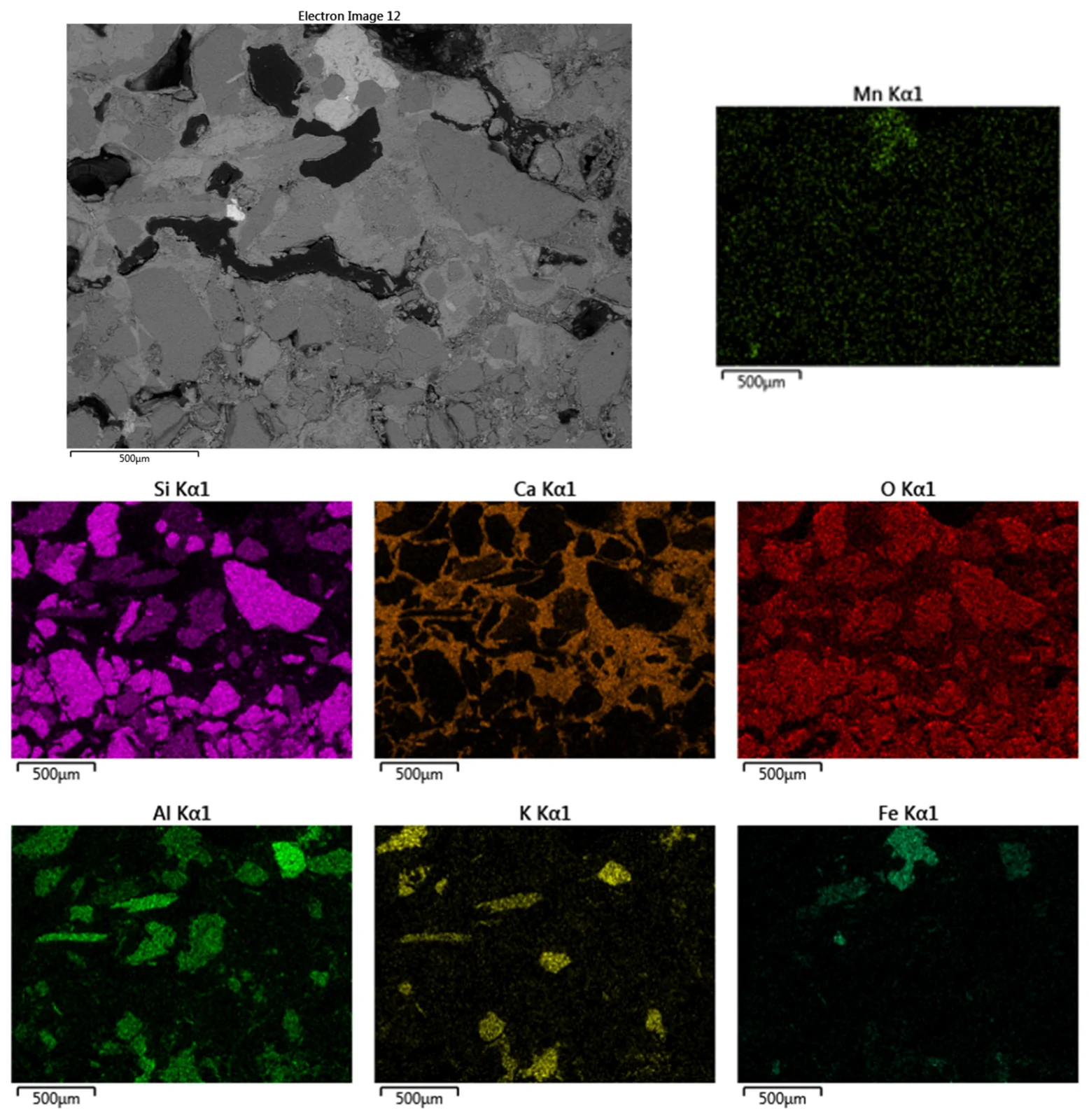
Elemental chemical composition map at the plaster/mortar interface.

The elemental chemical composition map of the plaster layer (Figure 7) shows that it contains a much lower proportion of silica (quartz sand particles) and a significant proportion of calcium, derived from hydrated lime. A clear variation in the Si/Ca ratio and sand/lime distribution is observed at the mortar–plaster interface.

**Figure 8:**
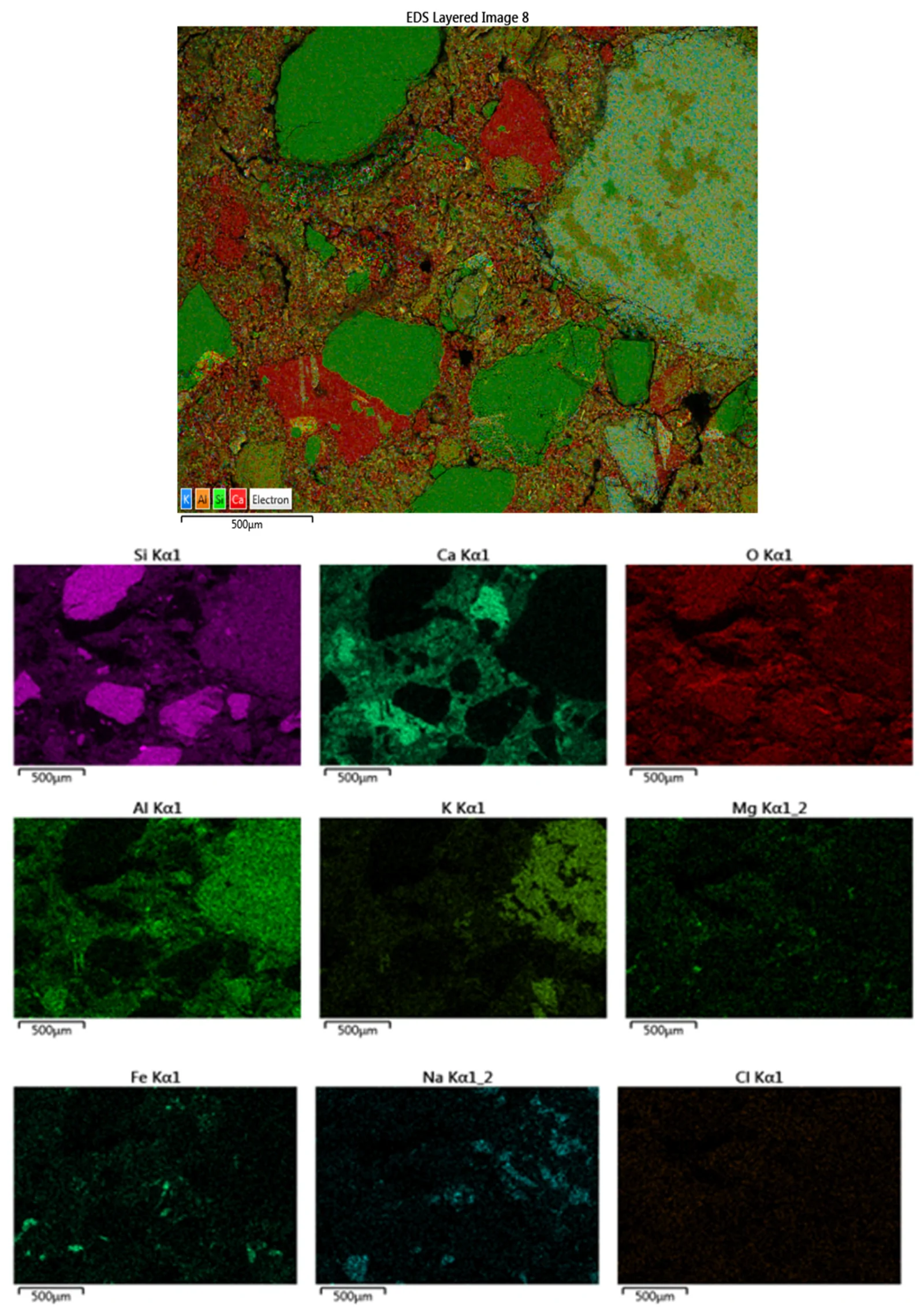
Elemental chemical composition map of the masonry mortar.

The masonry mortar exhibits a morphology characterized by a much higher proportion of sand and larger particle sizes. Large particles, generally rounded, consist of silica, lime, and other mineral components embedded within a calcium-rich matrix (Figure 8). These features highlight the structural differences between the coarse masonry mortar and the finer, protective plaster layer.

**Figure 9:**
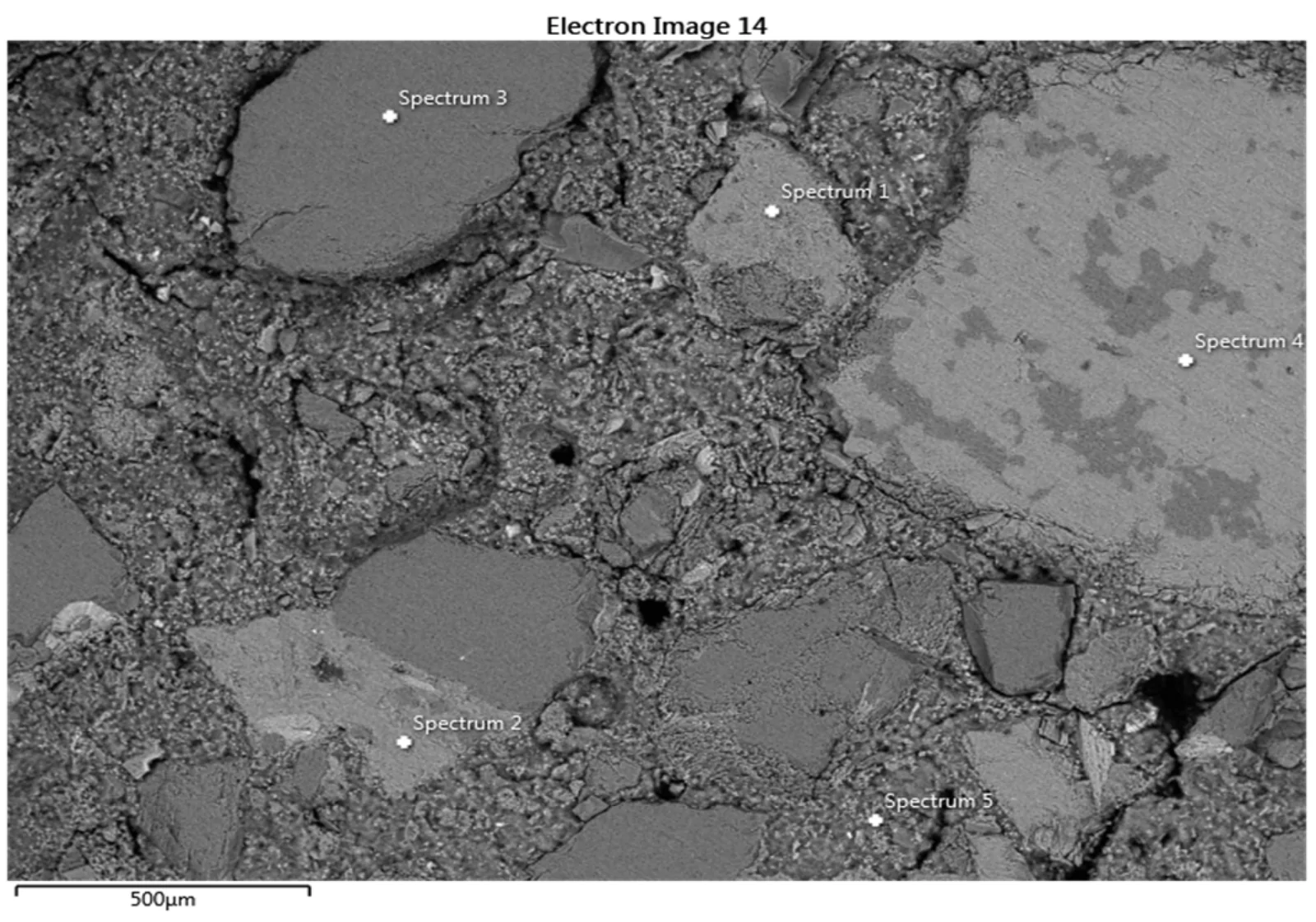
Five-point spectral analysis of the sample shown in Figure 8: 1,2—lime particles; 3,4—sand particles; 5—matrix.

The elemental chemical composition map of the masonry mortar shows the distribution of key components within the material. Point spectral analysis of calcium-rich particles, which are relatively small, and of the surrounding matrix is presented in Figure 9.

The calcium-rich particles are predominantly composed of calcium, with minor proportions of Al, Mg, K, Na, Fe, and S. The sand aggregates contain aluminum and potassium. The calcium content in the matrix is comparable to the silicon content (Figure 9, Table 2 and Table 3), reflecting the balanced composition of the mortar and the formation of reactive hydraulic phases contributing to long-term strength and durability.

**Table 2:** Chemical composition determined by SEM-EDS obtained by 5 point-scans presented in Figure 9.

Spectrum Label (wt%)	Spectrum 1	Spectrum 2	Spectrum 3	Spectrum 4	Spectrum 5
O	47.59	52.37	51.63	48.57	48.69
Na	0.27	0.20	-	0.35	0.26
Mg	0.38	0.45	-	-	0.67
Al	1.64	1.45	0.27	9.87	5.14
Si	5.75	6.15	47.55	29.64	19.85
S	0.36	0.16	-	-	0.19
Cl	-	-	-	-	0.23
K	0.69	-	-	11.57	2.00
ca	42.59	38.48	0.54	-	21.24
Fe	0.74	0.73	-	-	1.72
Total	100.00	100	100.00	100.00	100.00

**Table 3:** Chemical composition determined by SEM-EDS on the lime particle presented in Figure 10.

Spectrum Label	O	Na	Mg	Al	Si	P	Cl	K	Ca	Ti	Fe	Total
Map Sum Spectrum (wt%)	53.63	0.52	0.38	3.02	33.37	1.20	0.07	0.71	5.70	0.12	1.27	100.00

**Figure 10:**
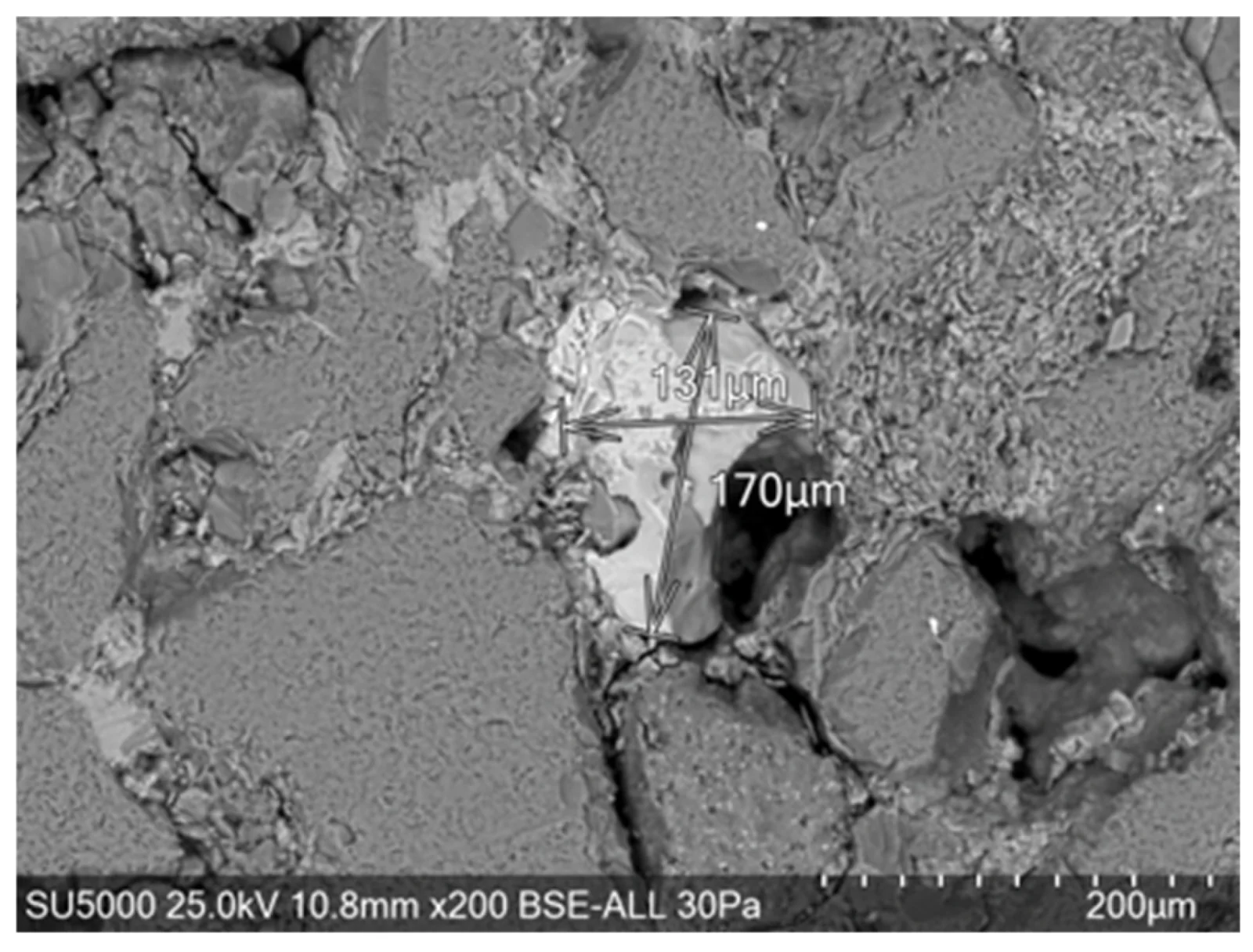
Microstructure and elemental chemical composition of a mortar area containing a lime particle (130 µm × 170 µm).

**Figure 11:**
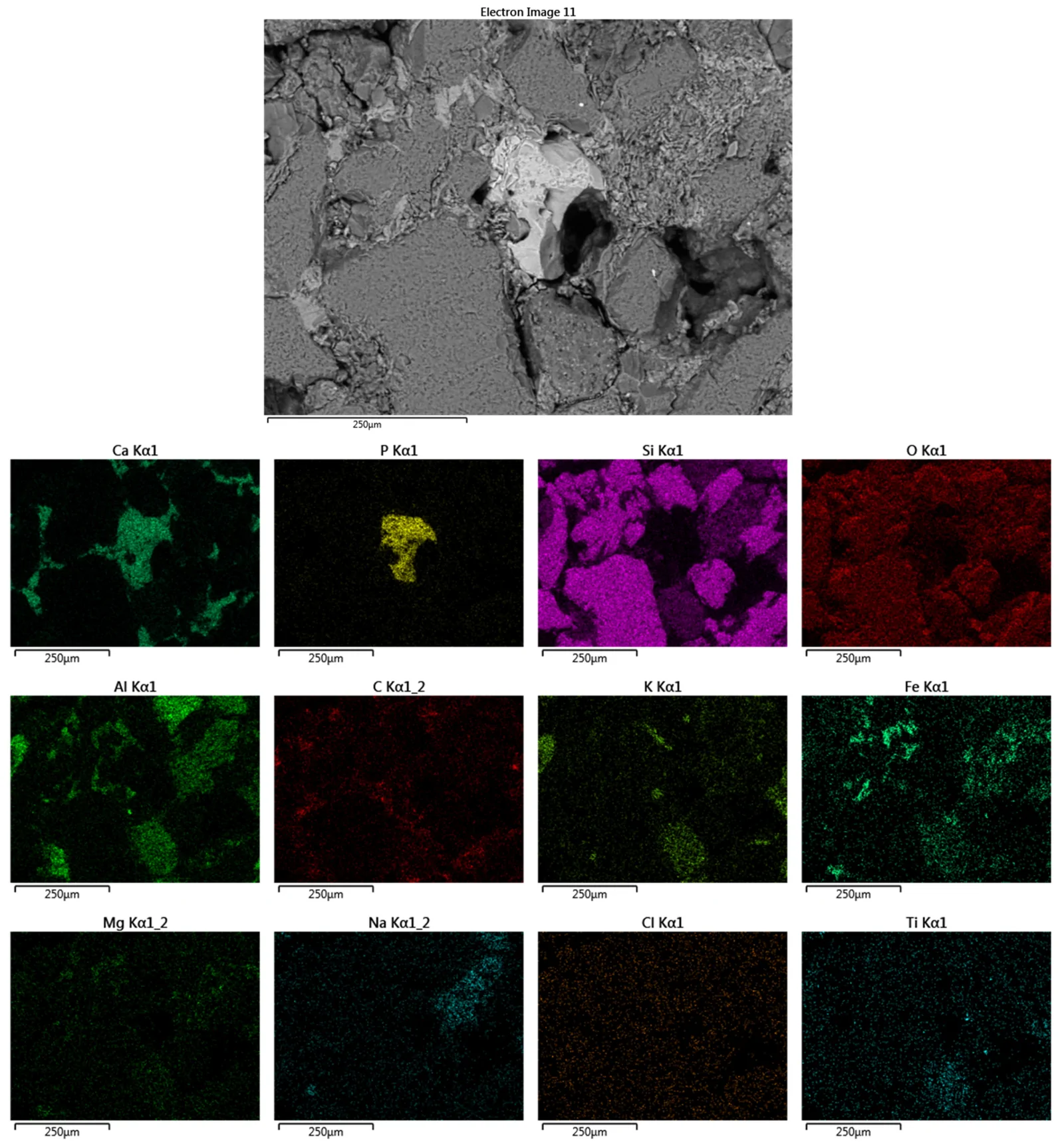
Elemental chemical composition map of the mortar in the area containing the lime particle.

**Figure 12:**
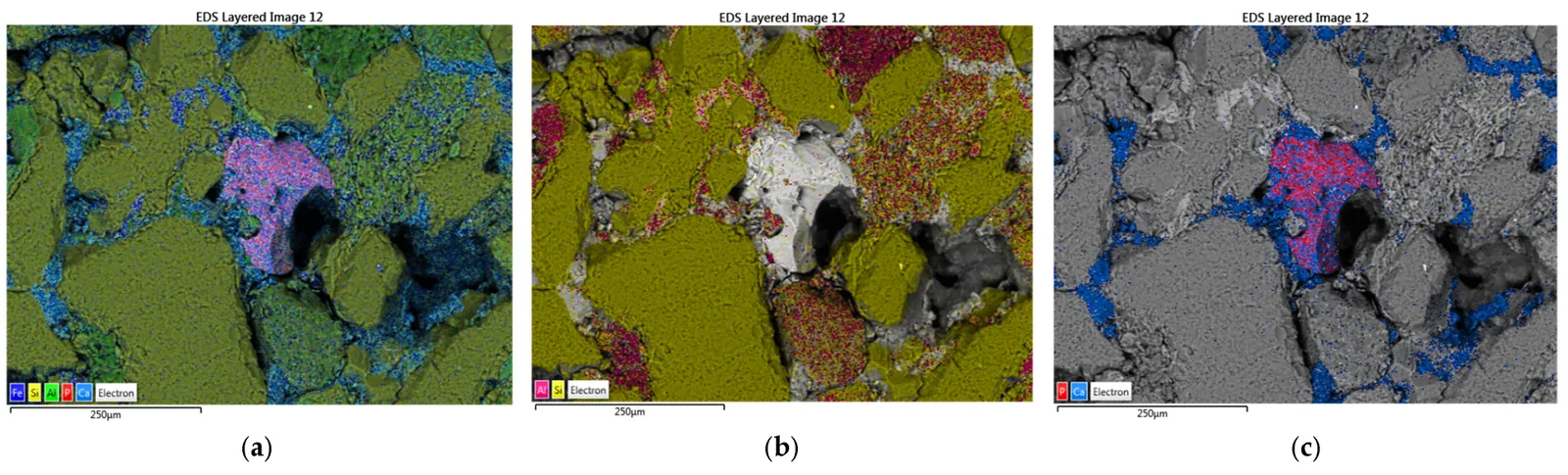
Distribution of major elements in the area of the lime particle (130 µm × 170 µm); (**a**) Fe, Si, Al, P, Ca distribution highlight; (**b**) Al, Si distribution highlight; (**c**) P, Ca distribution highlight.

Elemental chemical analysis was performed on a surface containing a large white particle (130 µm × 170 µm) to determine its composition (Figure 10) and the corresponding elemental composition map (Figure 11). In the large white particle, significant amounts of calcium and phosphorus are observed, with a relatively uniform distribution (Figure 12). In contrast, phosphorus was not detected in the smaller lime particles.

**Figure 13:**
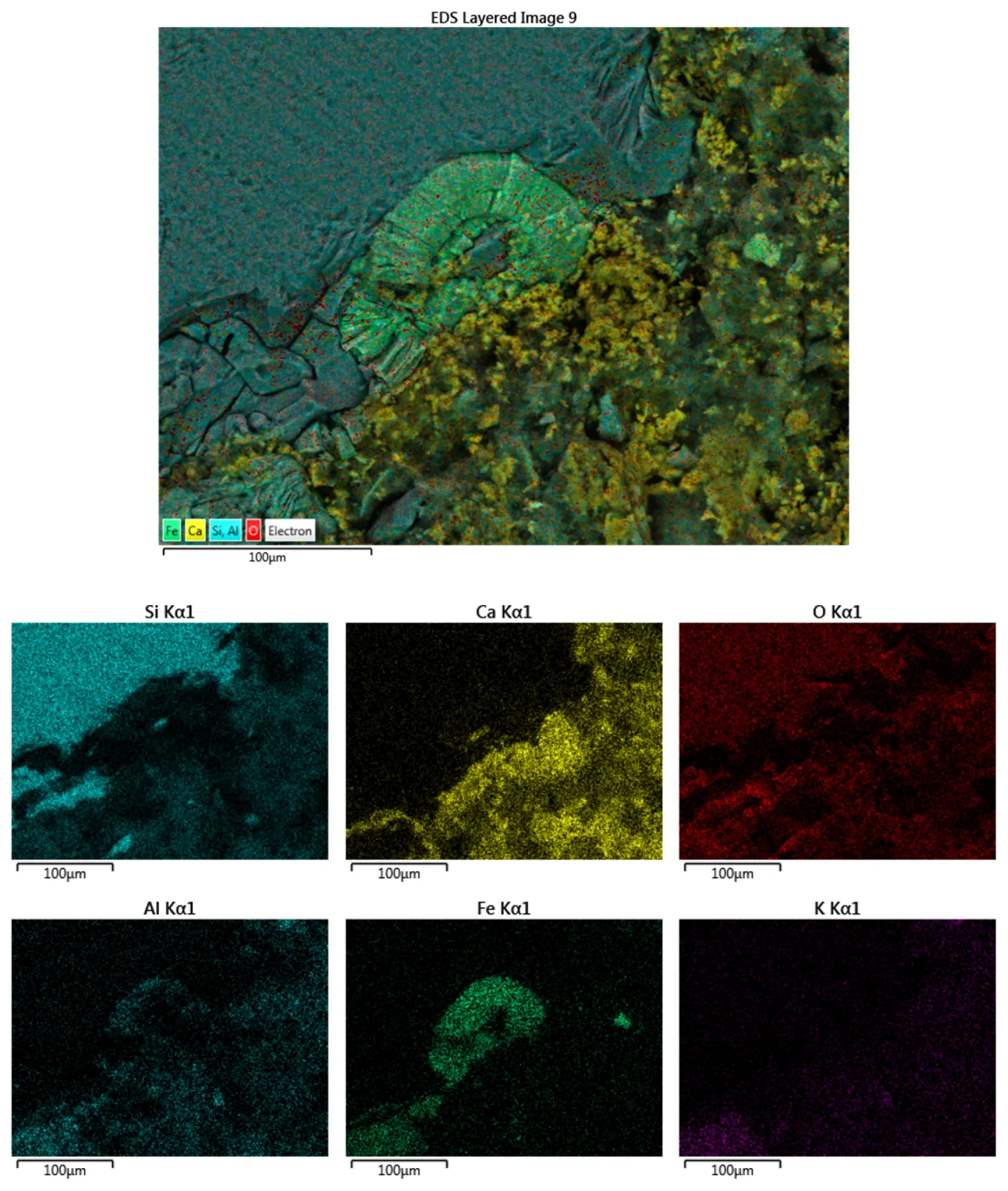
Elemental chemical composition map of the mortar in an area with iron-rich particles.

**Figure 14:**
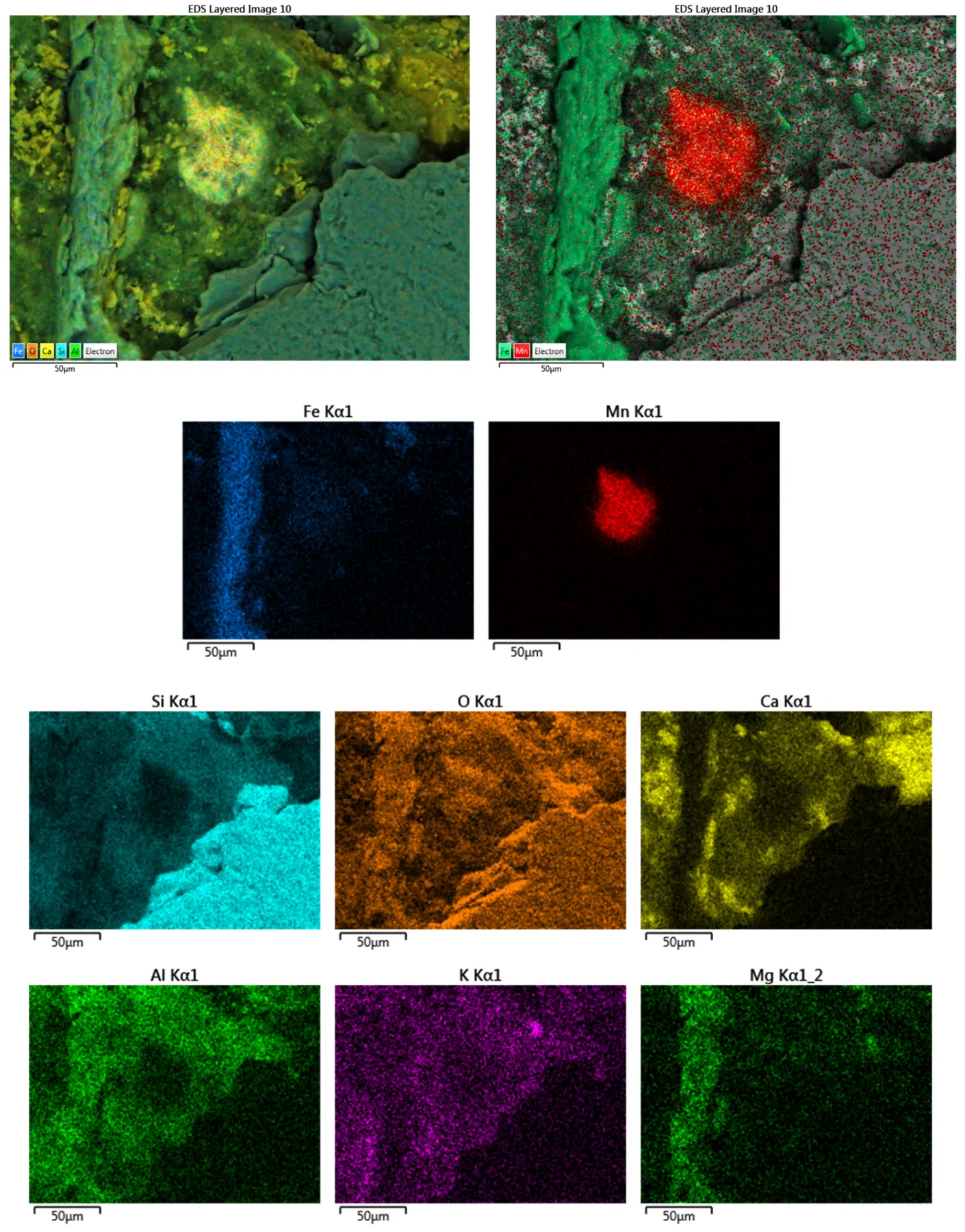
Elemental chemical composition map in an area with Fe- and Mn-rich particles.

**Figure 15:**
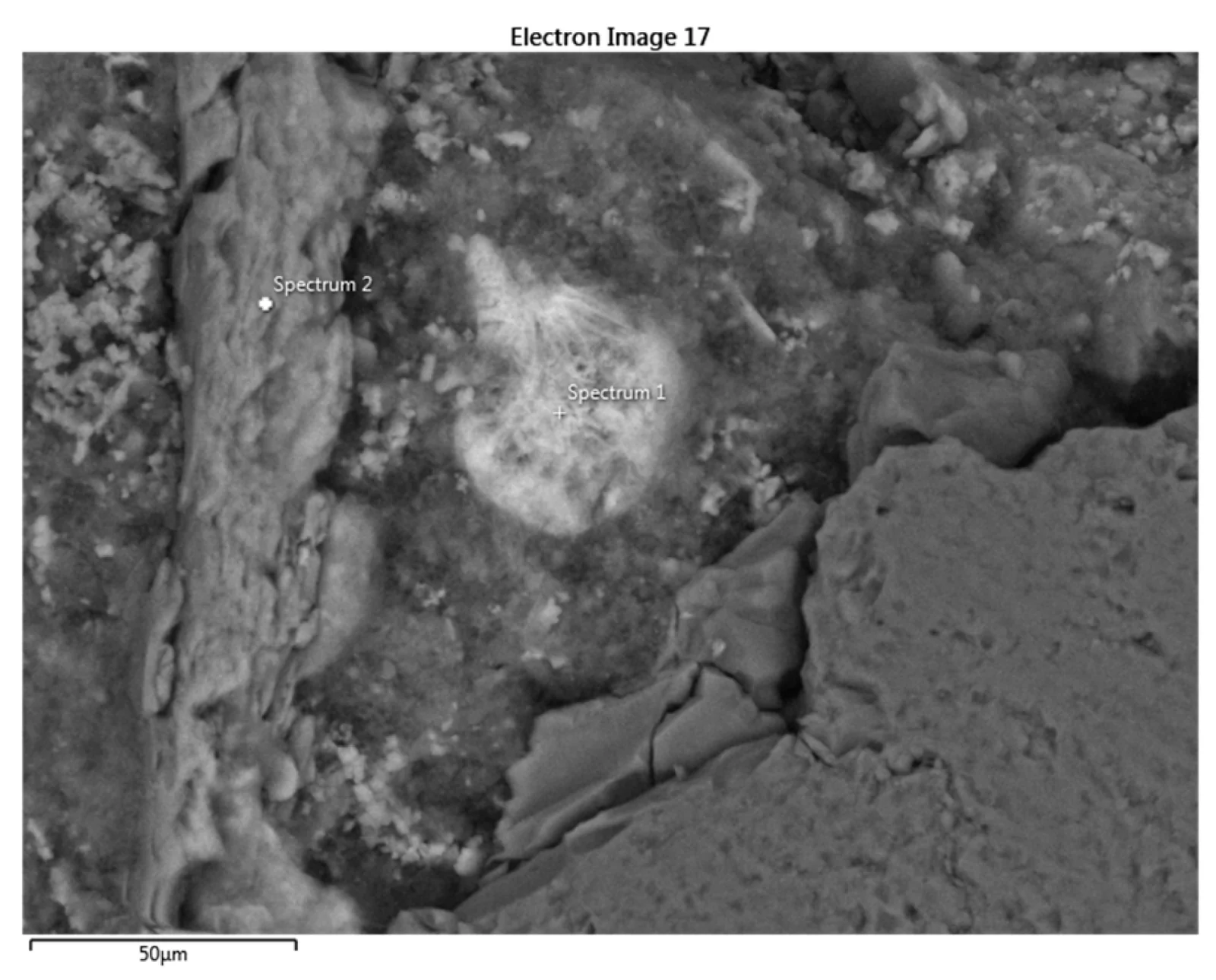
Point scan analysis of the area shown in Figure 14.

**Figure 16:**
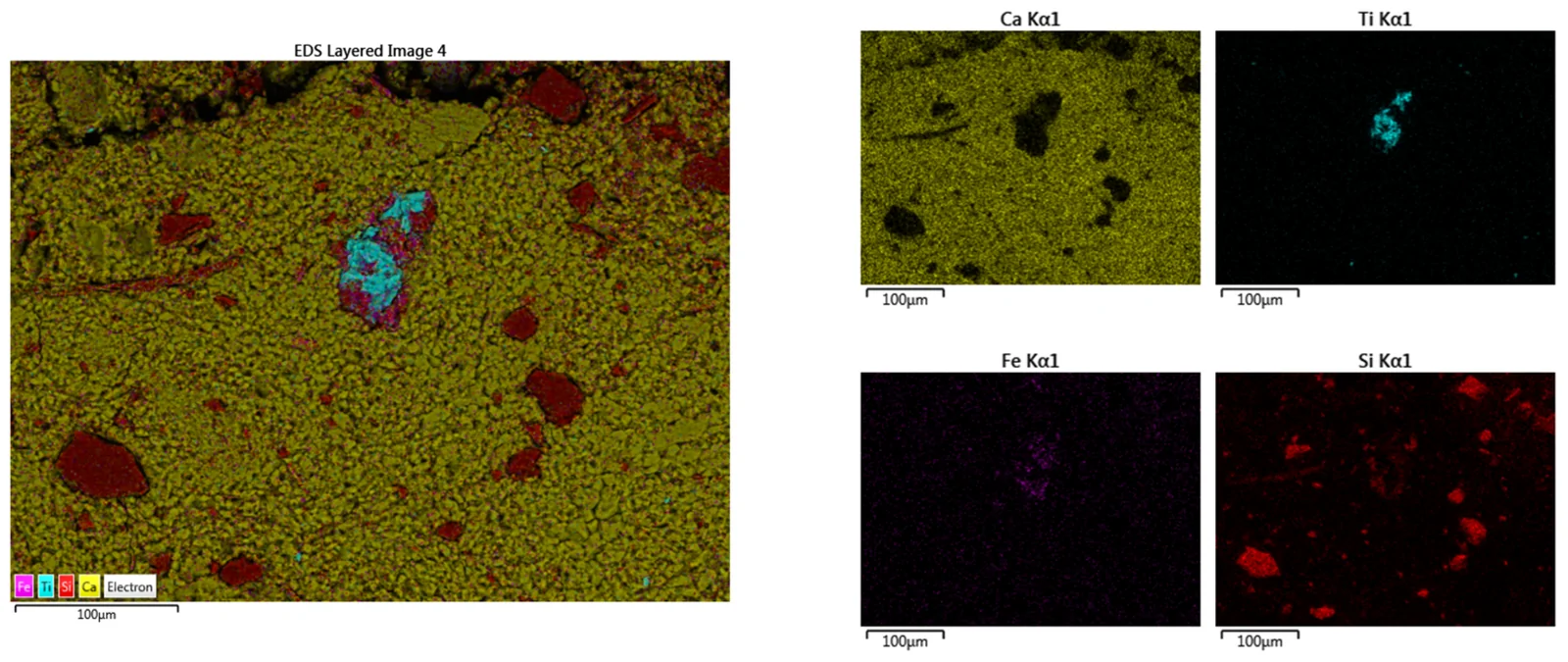
Elemental chemical composition map showing Ti- and Fe-rich particles in the mortar matrix.

In the mortar matrix, large particles highly enriched in iron and manganese were identified (Figure 13, Figure 14 and Figure 15), as well as particles containing titanium (Figure 16).

Point spectral analysis of the metal-rich area (Figure 14, Table 4) shows the following composition: particle 1 contains 38% Mn, 2% Al, and 1.75% Fe, while particle 2 contains 21.53% Fe and 8.42% Al. These metal-rich particles are embedded within the calcium-rich mortar matrix and contribute to the heterogeneity of the material, potentially affecting mechanical properties and long-term durability.

Point scan analysis was performed on the metal-rich particles. Particle 1 is enriched in manganese (Mn), while particle 2 is enriched in iron (Fe).

**Figure 17:**
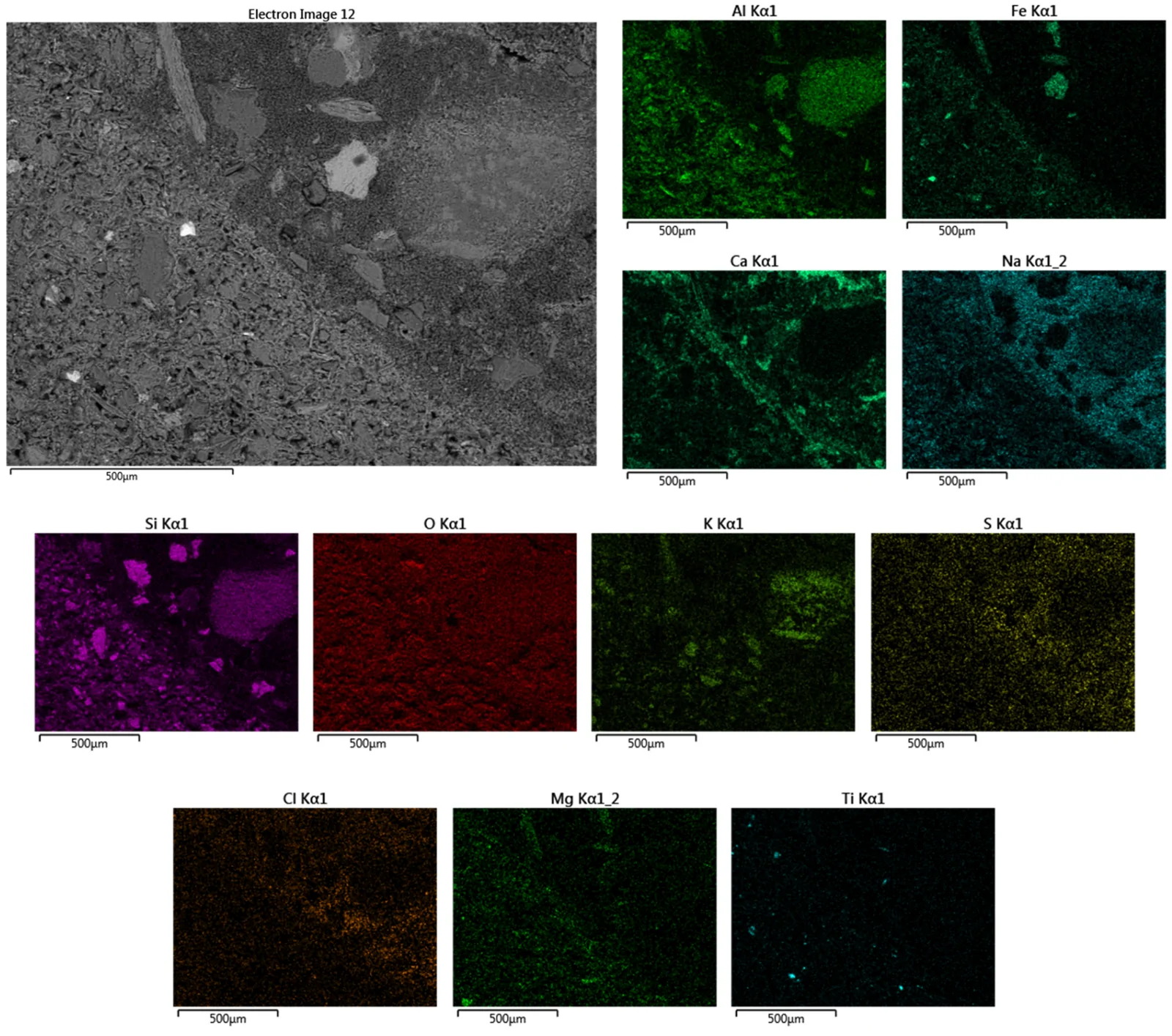
Elemental chemical composition map of the interface area between masonry mortar and plaster mortar.

SEM-EDS characterization of an interface area between the masonry mortar and the reddish-brown plaster layer highlights an excellent quality of the interface and microstructural and chemical composition differences between the two mortars (Figure 17). At the interface, a very good bonding between the plaster and masonry mortar was observed, with no discontinuities, compaction defects, or cracks detected.

The elemental composition map (Figure 17) of the interface area shows distinct differences between the two materials: variations in particle composition, element distribution within the calcium-rich matrix, the presence of Al- and Fe-rich components, and traces of sulfur. These observations confirm the high quality of the interface and the compatibility of the plaster with the underlying masonry mortar.

**Table 4:** Chemical composition determined by SEM-EDS obtained by 2 point-scans presented in Figure 15.

Spectrum Label (wt%)	Spectrum 1	Spectrum 2
O	40.06	45.71
Na	0.26	-
Mg	1.50	5.41
Al	2.06	8.42
Si	6.63	13.95
K	0.56	0.92
Ca	3.16	3.38
Ti	-	0.69
Mn	38.95	-
Fe	1.75	21.53
Ba	5.05	-
Total	100.00	100.00

**Figure 18:**
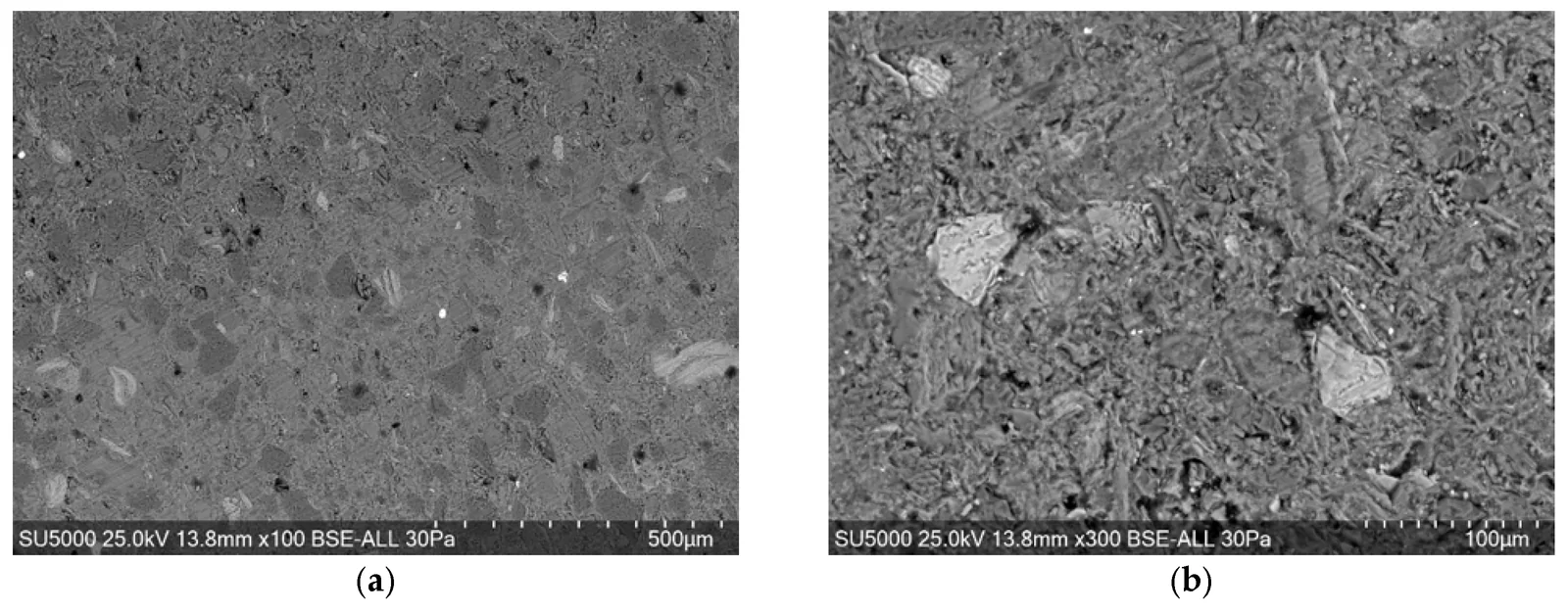
Microstructure of the reddish-brown plaster mortar sample at different magnifications (**a**) ×100; (**b**) ×300.

**Figure 19:**
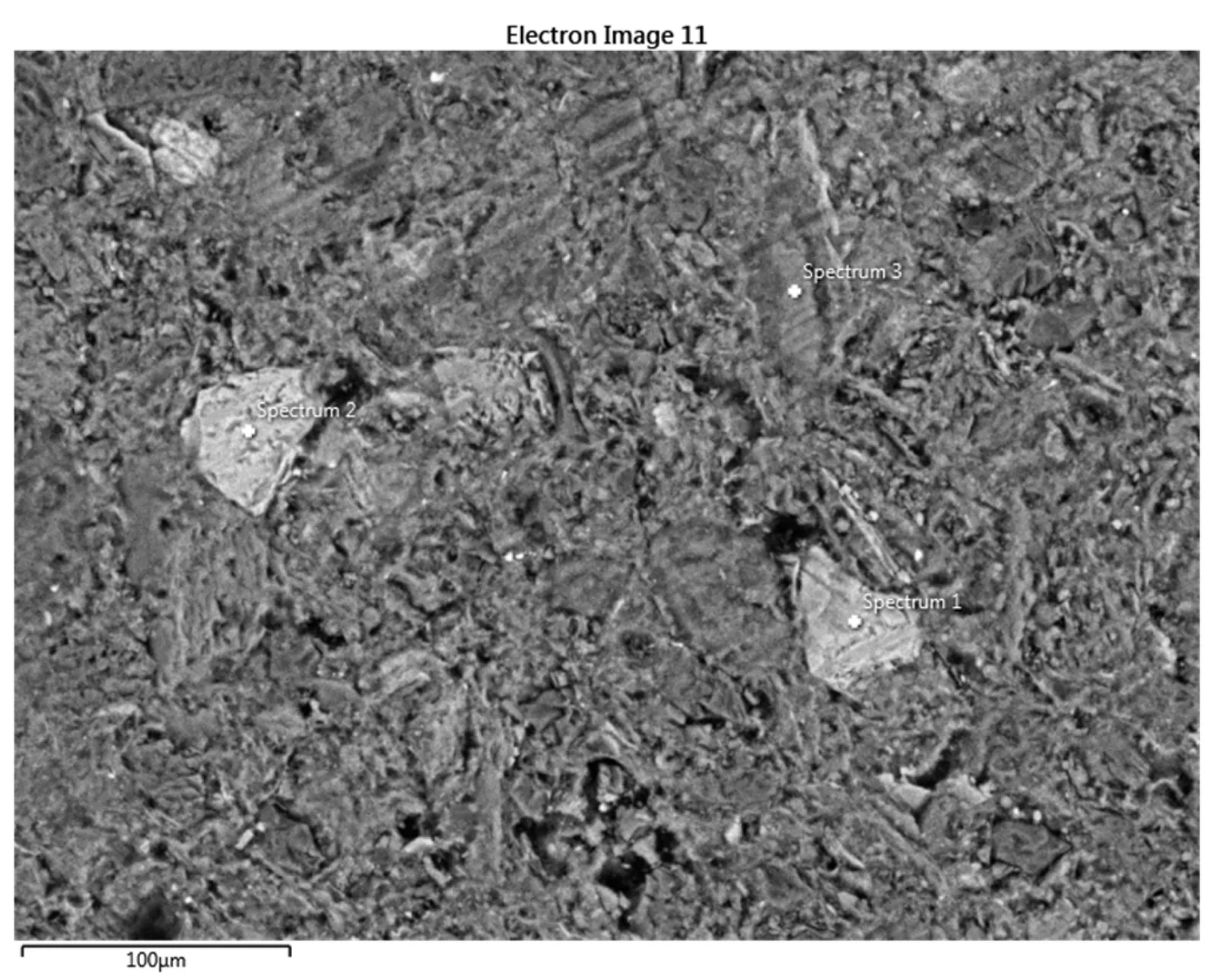
Three-point spectral analysis of the plaster mortar.

The structure and composition of the reddish-brown material are typical of a mortar, consisting of a calcium-rich matrix, siliceous particles, and other inclusions. The reddish-brown color, resulting from the addition of brick powder, and its strong adhesion to the underlying masonry mortar indicate that this is a plaster mortar corresponding to the Roman designation Opus signinum, a fine, reddish plaster or flooring mortar. The plaster mortar exhibits a fine texture with a slightly acidic appearance and contains white particles (Figure 18 and Figure 19, Table 5).

The plaster mortar exhibits a fine-grained structure with white inclusions dispersed within a calcium-rich matrix, reflecting its protective and finishing function.

Point spectral analysis of the plaster mortar shows a high lime content in the matrix (point 3) and elevated phosphorus levels in the large white particles (points 1 and 2). This distribution indicates the presence of reactive phosphorus-bearing phases that may enhance the hydraulic behavior and contribute to the self-healing capacity of the plaster mortar.

**Figure 20:**
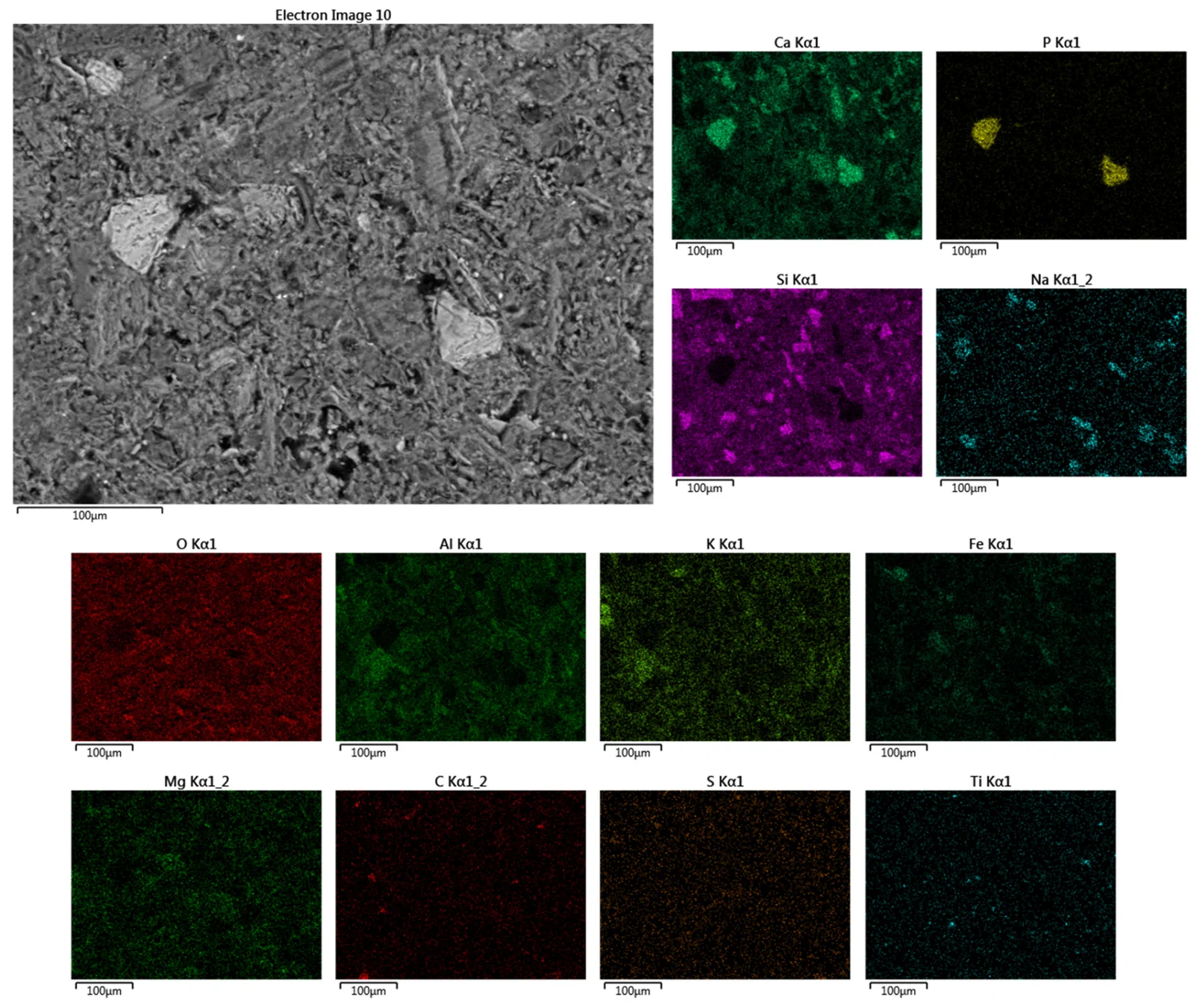
Elemental chemical composition map of the reddish plaster mortar.

The elemental composition map of the plaster mortar area (Figure 20) shows that only the large calcium-rich particles contain phosphorus.

**Figure 21:**
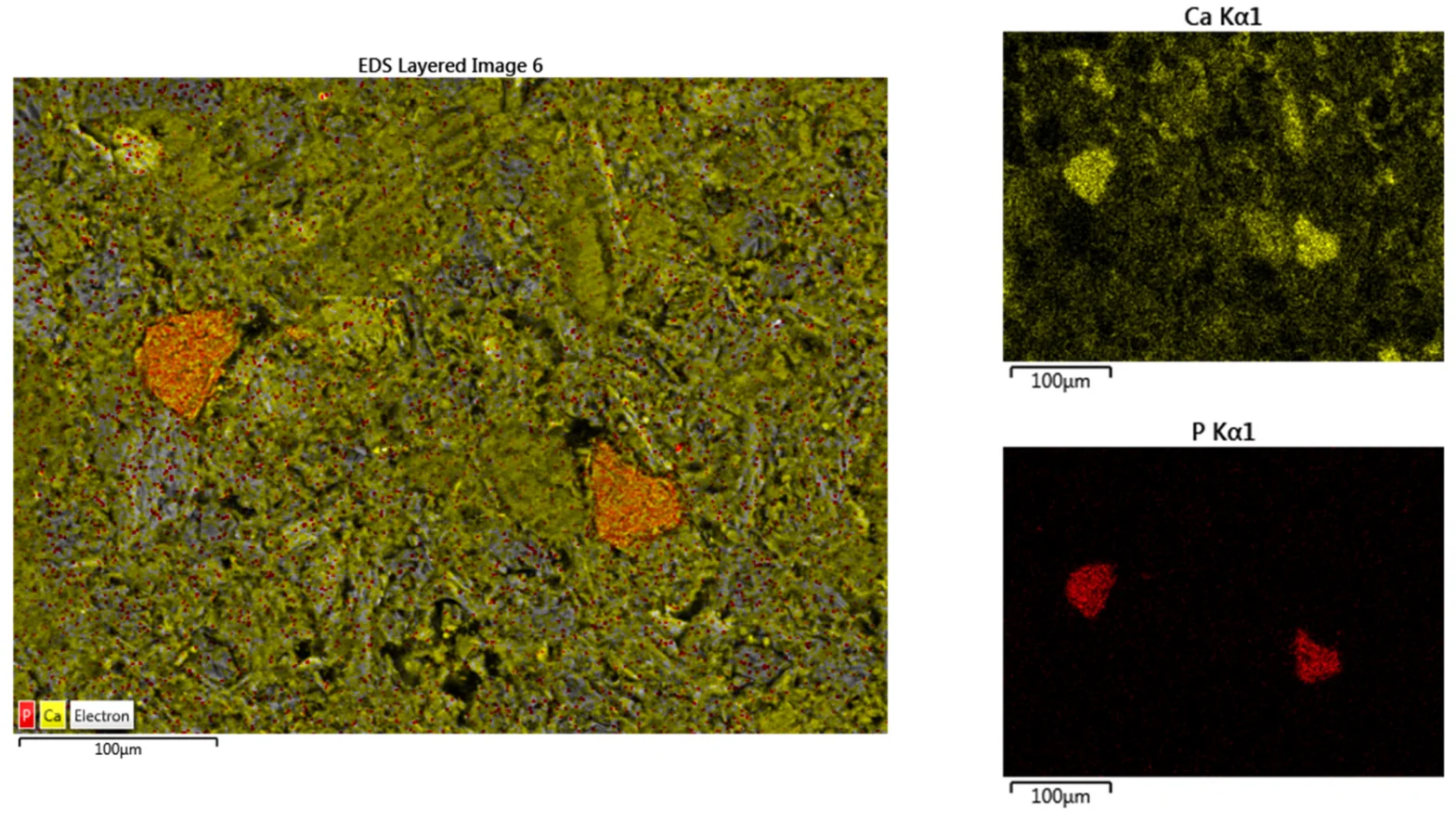
Distribution of calcium and phosphorus in the white particles.

The distribution of calcium and phosphorus (Figure 21) is similar to that observed in the masonry mortar analyzed previously (Figure 8, Figure 9, Figure 11 and Figure 12).

Small calcium-rich particles do not contain phosphorus and are evidently lime particles. The large white particles exhibit a high phosphorus content.

The evaluation of the possible occurrence of phosphorus-rich limestone deposits in the region, as well as the potential use of limestone deposits located on the Danube terraces for lime production, indicates that these sources could not account for the high phosphorus concentrations observed [45,46,47]. Large white particles exhibit a calcium-to-phosphorus (Ca/P) ratio similar to that of bone. The elemental chemical composition maps show that calcium (Ca) and phosphorus (P) are co-localized within a particle whose morphology is consistent with that of a calcined bone fragment. The presence of bone fragments remaining in the ash after combustion indicates that bone ash was intentionally used in the preparation of the mortar [48,49]. Bone ash is not mentioned by Vitruvius in the Roman mortar recipe. Knowing that in this part of the Roman Empire builders did not have access to volcanic pozzolanic ash and that Romans primarily used locally available materials, we consider that the Sucidava mortar included an addition of bone ash, whose composition is dominated by phosphates, not silicates as in volcanic ash [50,51].

**Figure 22:**
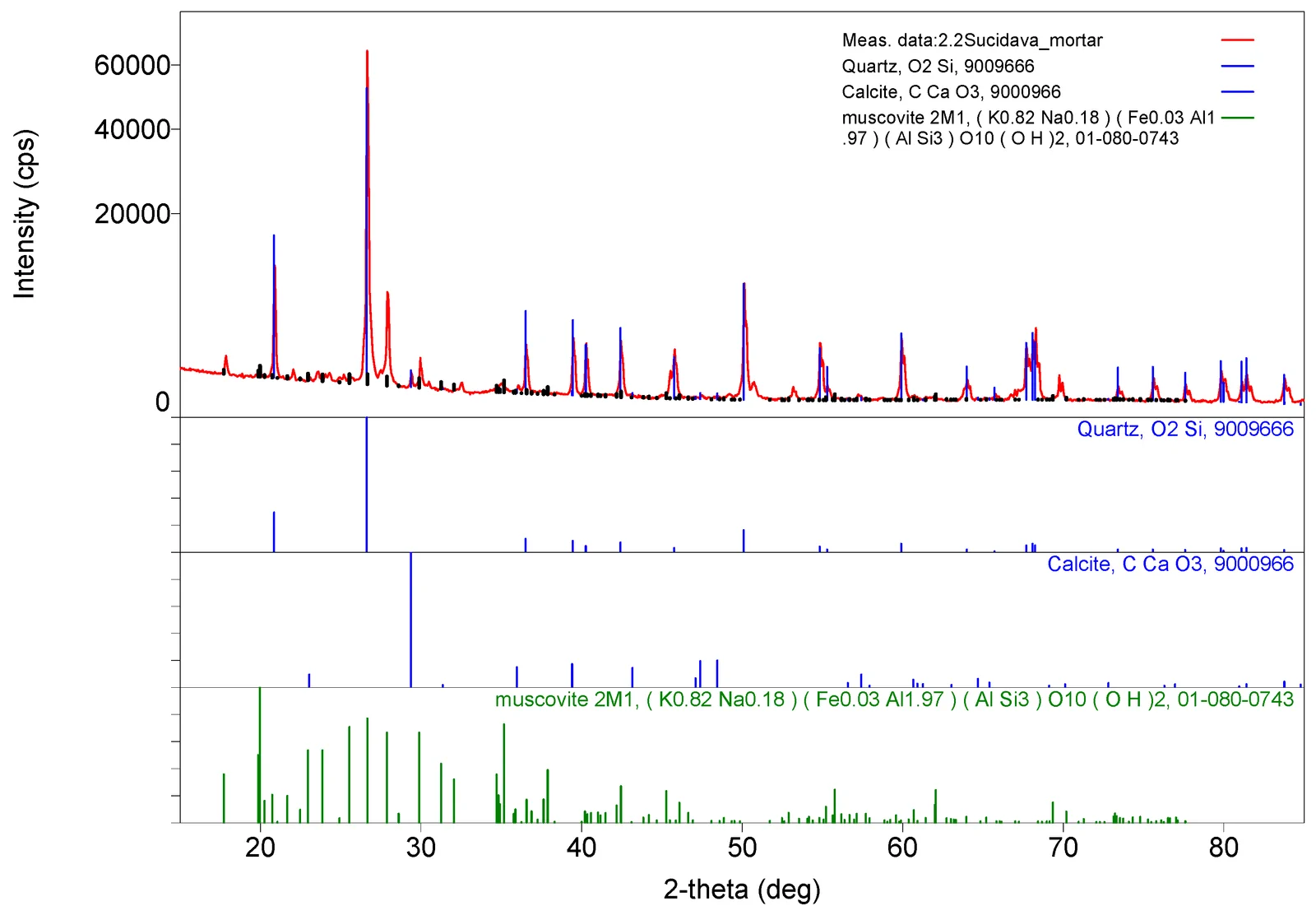
X-ray diffraction (XRD) spectrum of the mortar sample with characteristics of the phases.

**Figure 23:**
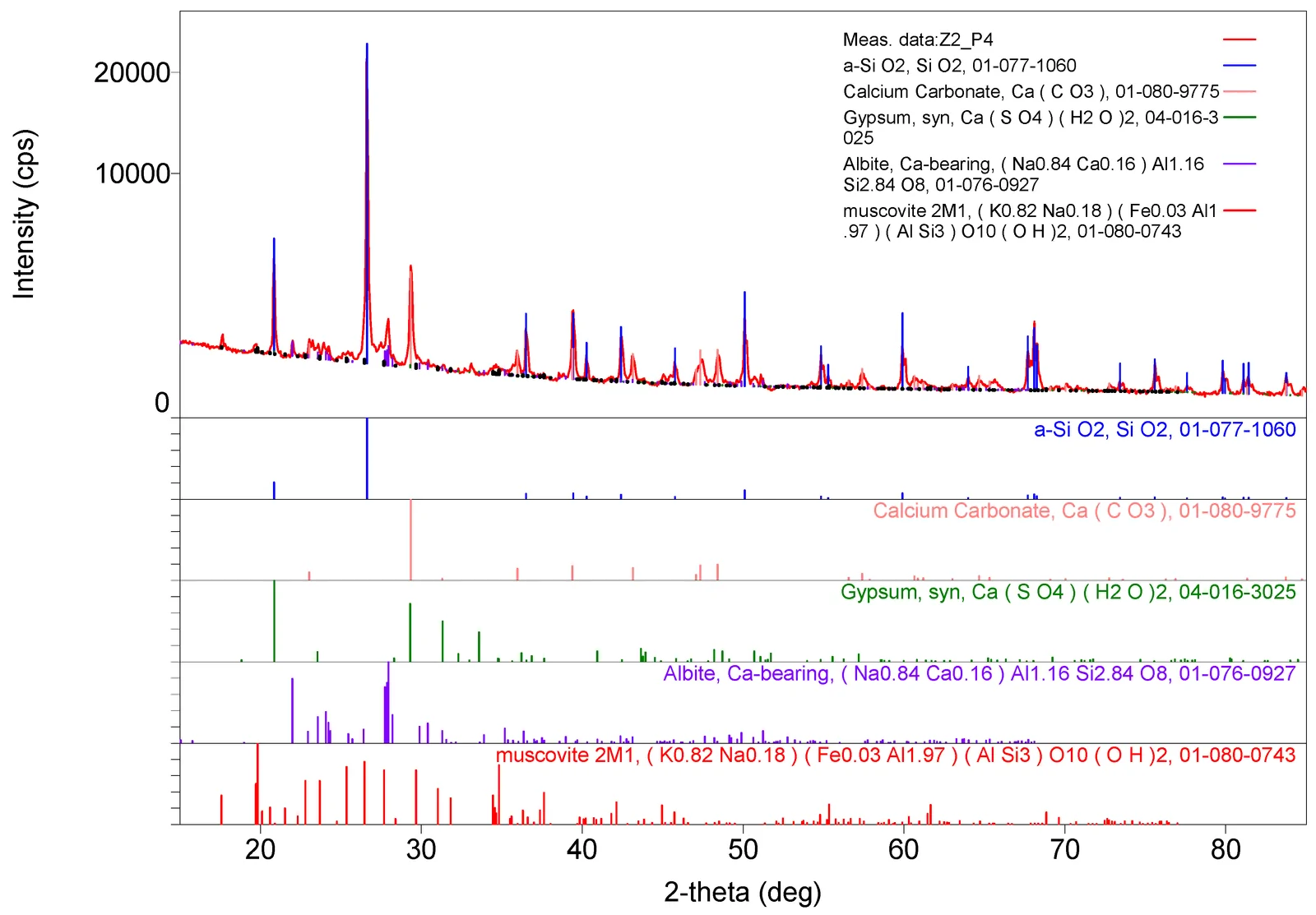
X-ray diffraction (XRD) spectrum of Z2_P4 with characteristics of the phases.

**Figure 24:**
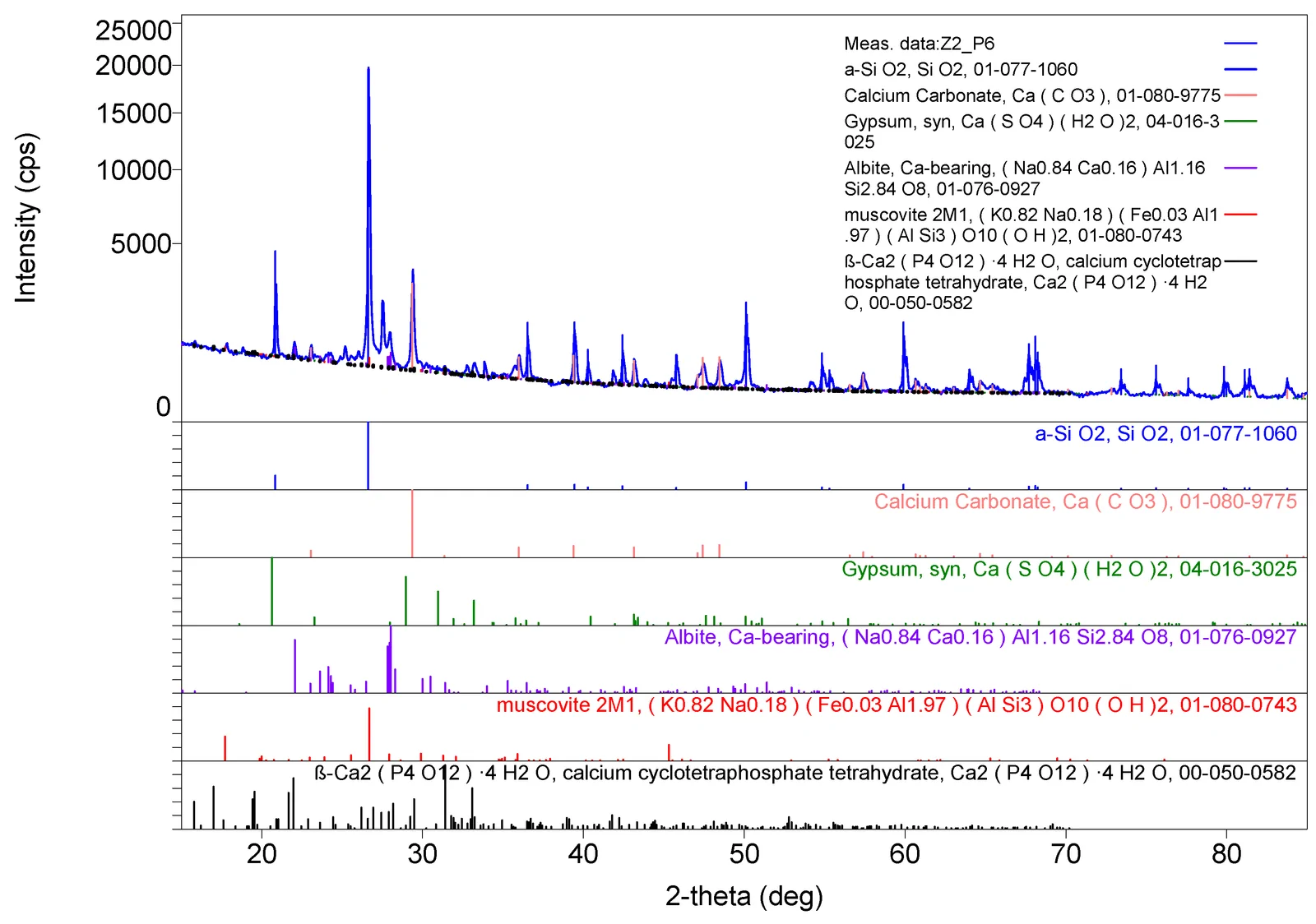
X-ray diffraction (XRD) spectrum of Z2_P6 with characteristics of the phases.

Qualitative phase analysis by X-ray diffraction (XRD) of the mortar samples (Figure 22, Figure 23 and Figure 24, Table 6, Table 7 and Table 8) highlighted the usual mortar phases (calcium carbonate/calcite and silica) and the presence of silicates, consistent with the theory proposed by Marie Jackson regarding Roman mortar [8,9,10,12,13,14].

Qualitative XRD phase analysis of the mortar.

**Table 6:** The characteristic phases of the mortar: quartz, calcite, and silicates.

Phase Name	Formula	Figure of Merit	Phase Reg. Detail	DB Card Number
Quartz	SiO_2_	0.499	ICDD (PDF-5+ 2026)	9009666
Calcite	CaCO_3_	1.545	ICDD (PDF-5+ 2026)	9000966
Muscovite 2M1	(K0.82 Na0.18) (Fe0.03 Al1.97) (Al Si3) O10 (OH)_2_	1.751	ICDD (PDF-5+ 2026)	01-080-0743

**Table 7:** Qualitative XRD phase analysis with identification of gypsum as a degradation product.

Phase Name	Formula	Figure of Merit	Phase Reg. Detail	DB Card Number
a-SiO_2_	SiO_2_	0.696	ICDD (PDF-5+ 2025)	01-077-1060
Calcium Carbonate	Ca (CO_3_)	0.489	ICDD (PDF-5+ 2025)	01-080-9775
Gypsum, syn	Ca (SO_4_) (H_2_O)_2_	1.879	ICDD (PDF-5+ 2025)	04-016-3025
Albite, Ca-bearing	(Na0.84 Ca0.16) Al1.16 Si2.84 O8	1.299	ICDD (PDF-5+ 2025)	01-076-0927
Muscovite 2M1	(K0.82 Na0.18) (Fe0.03 Al1.97) (Al Si3) O10 (OH)_2_	1.687	ICDD (PDF-5+ 2025)	01-080-0743

**Table 8:** Qualitative XRD analysis showing the presence of Ca_2_(P_4_O_12_)·4H_2_O corresponds to a hydrated calcium phosphate phase.

Phase Name	Formula	Figure of Merit	Phase Reg. Detail	DB Card Number
a-SiO_2_	SiO_2_	0.451	ICDD (PDF-5+ 2025)	01-077-1060
Calcium Carbonate	Ca (CO_3_)	0.567	ICDD (PDF-5+ 2025)	01-080-9775
Gypsum, syn	Ca (SO_4_) (H_2_O)_2_	2.944	ICDD (PDF-5+ 2025)	04-016-3025
Albite, Ca-bearing	(Na0.84 Ca0.16) Al1.16 Si2.84 O8	1.351	ICDD (PDF-5+ 2025)	01-076-0927
Muscovite 2M1	(K0.82 Na0.18) (Fe0.03 Al1.97) (Al Si3) O10 (OH)_2_	1.842	ICDD (PDF-5+ 2025)	01-080-0743
ß-Ca2 (P4 O12)·4 H_2_O, calcium cyclotetraphosphate tetrahydrate	Ca2 (P4 O12)·4 H_2_O	1.739	ICDD (PDF-5+ 2025)	00-050-0582

The effect of Ca–P–Si compounds on the properties of hydraulic mortars manifests as increased hydraulic reactivity. Calcium silicates (C_2_S, C_3_S) react with water to form C–S–H gel (calcium silicate hydrate) and Ca(OH)_2_ [8,9,10,12,13,14,52].

The C–S–H (calcium silicate hydrate) and C–A–S–H (calcium alumino-silicate hydrate) gels are not stoichiometric compounds but rather amorphous or poorly crystalline gels with variable compositions. C–S–H gels consist primarily of CaO, SiO_2_, and H_2_O. Their general formula can be expressed as Ca_x_SiO_2+x_·nH_2_O. C–A–S–H gels have been identified as the products of aluminum incorporation, originating from volcanic ash (pozzolan), into the C–S–H structure. These gels contain CaO, SiO_2_, Al_2_O_3_, and H_2_O, and may also include small amounts of MgO, Fe_2_O_3_, Na_2_O, and K_2_O. The modifications introduced into the composition of these gels through the use of bone ash and local aggregates require a comprehensive investigation. The presence of phosphates modifies the network, increases reactivity, and leads to the formation of complex hydrated phases (C–S–P–H). The result is faster setting and higher initial strength. The superior mechanical strength of phosphorus-containing mortars is explained by the silicate–phosphate phase providing a dense microstructure with stronger bonds. Fine particles from bone ash act as a filler material, reducing porosity, filling spaces between sand and lime particles, and making the structure more compact. The formation of Ca–P–Si hydrated compounds improves intergranular adhesion. Compressive strength and freeze–thaw resistance are better than in simple mortars or those doped only with HAp. By modifying the microstructure and mineral composition, the mortar can become more resistant to moisture and degradation [53,54,55,56].

**Figure 25:**
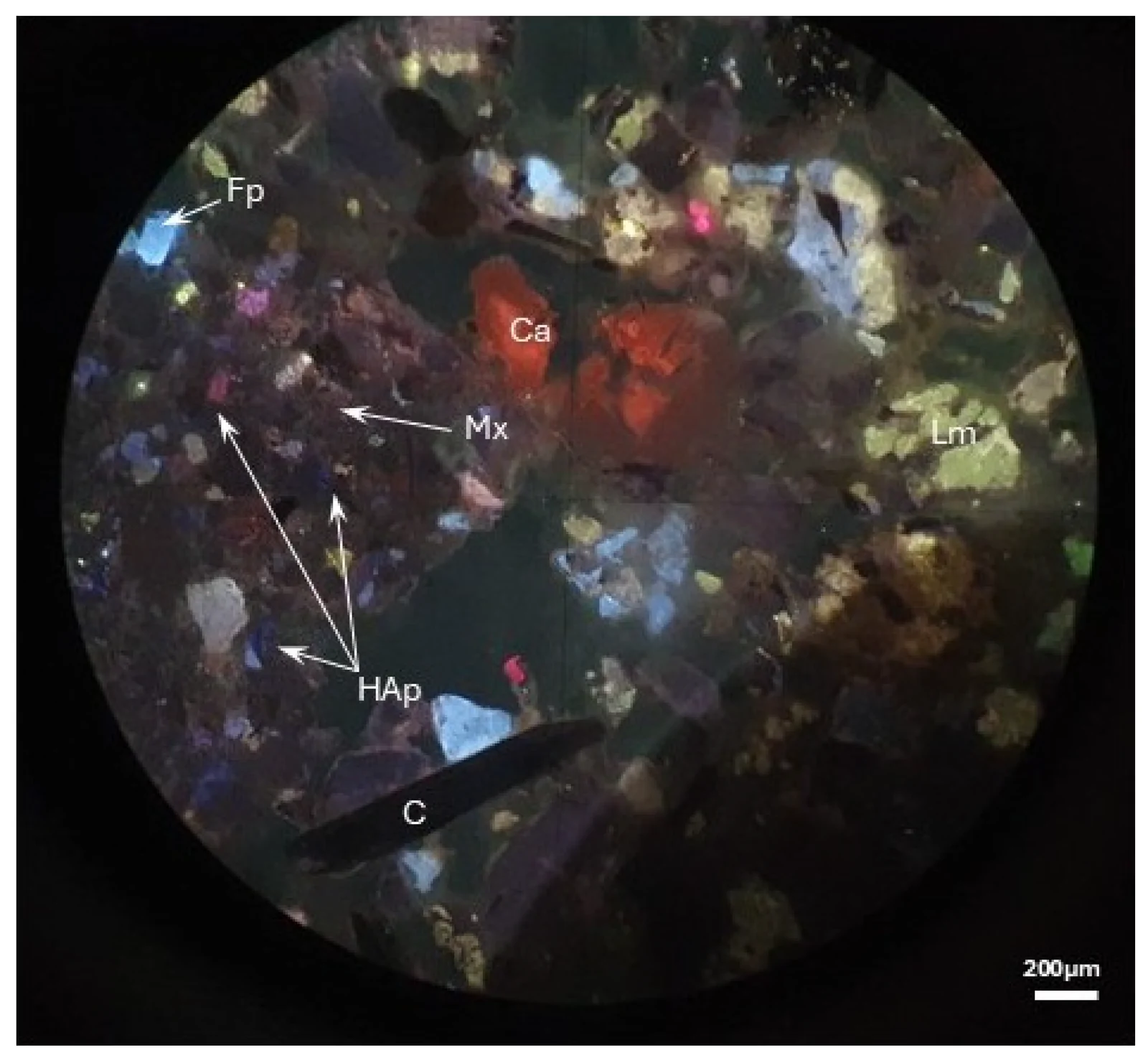
Cathodoluminescence microscope image: Mx—matrix with diffuse luminescence, brown-violet in color (possibly containing amorphous hydroxyapatite); HAp—fine hydroxyapatite fragments, violet in color, with low luminescence; Fp—feldspar fragments; Ca—calcite; Lm—metamorphic lithoclasts; C—carbonized plant fragments.

The elemental chemical composition maps show that Ca and P are co-localized within a particle exhibiting a morphology consistent with that of a calcined bone fragment. The large white particles, analyzed by SEM–EDS, exhibited a lower calcium content and a higher phosphorus content. This can be explained by the initial presence of calcium-deficient carbonated hydroxyapatite, the formation of β-calcium phosphate, the loss of part of the calcium compounds during bone burning, changes in calcium distribution in the presence of impurities (Na, K, Mg, and Cl), and the limitations of the EDS analytical method, which quantifies light elements with lower accuracy and may underestimate or overestimate the Ca/P ratio. The X-ray diffraction (XRD) identified the presence of β-calcium pyrophosphate tetrahydrate, β-Ca_2_(P_4_O_12_)·4H_2_O (Figure 25), which may have formed as a result of bone burning [57,58,59,60,61].

**Figure 26:**
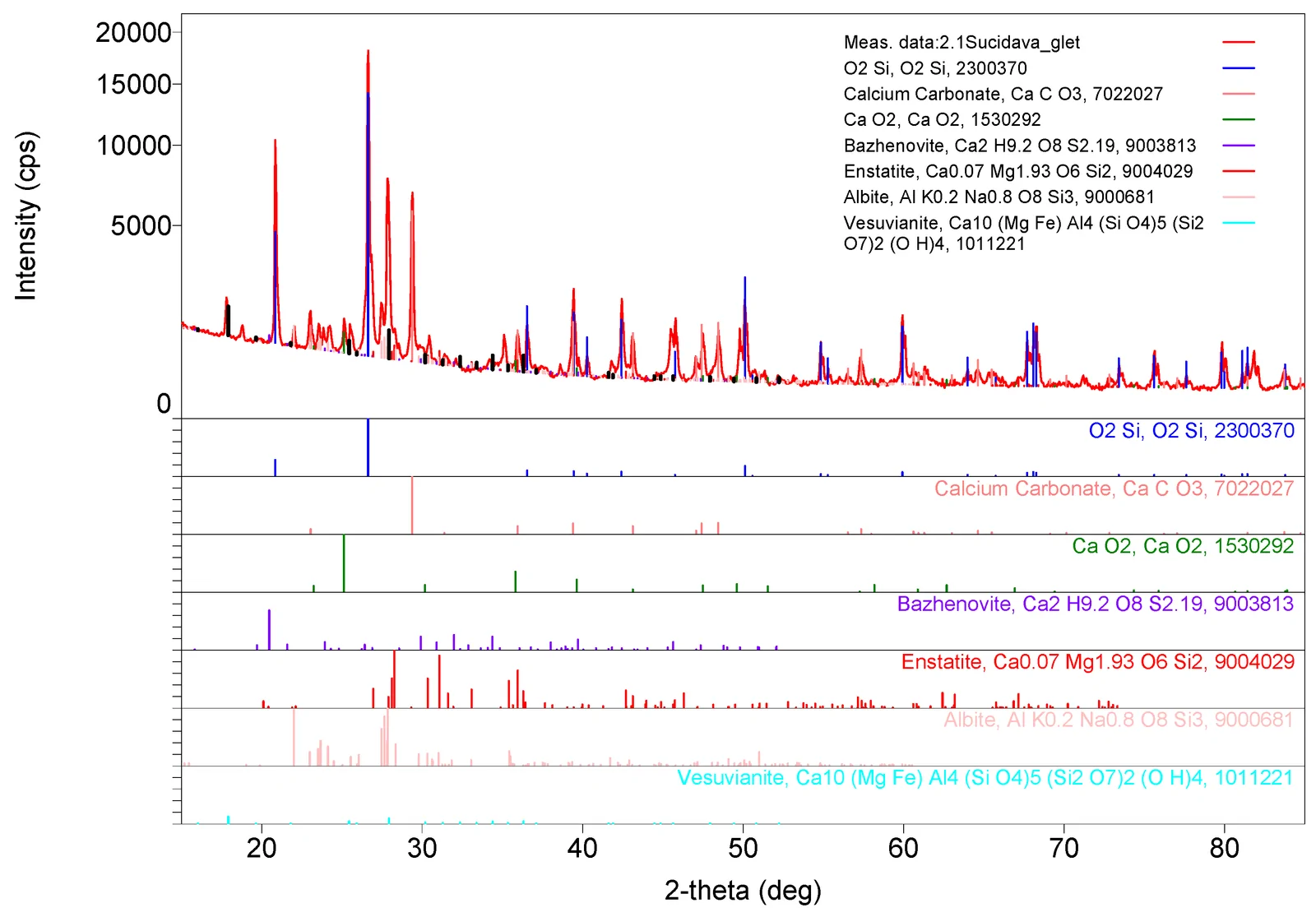
X-ray diffraction (XRD) spectrum of the plaster mortar with characteristics of the phases.

The identification of the crystalline phases Bazhenovite, Vesuvianite, and CaO_2_ should be interpreted with caution. Bazhenovite is a rare mineral phase and has not been commonly reported in Roman mortars. Therefore, its identification is considered tentative and will require confirmation by complementary analytical techniques. The occurrence of Vesuvianite is more plausible. Roman builders frequently exploited locally available raw materials with favorable engineering properties. Consequently, the mineralogical composition of the aggregates could be relatively complex. In Roman mortars, the presence of Vesuvianite may reflect the use of aggregates derived from Vesuvianite-bearing rocks, reactions between lime and aluminosilicate impurities, or the incorporation of volcanic or metamorphic materials. Regarding the phase initially identified as CaO_2_, we consider that this assignment is unlikely because calcium peroxide is a highly unstable compound and would not be expected to persist in a mortar approximately 1600 years old. A more plausible interpretation is that the detected reflections correspond to CaO (quicklime)**,** which was intentionally used by Roman builders during mortar preparation through the hot-mixing technique. Recent research on Roman mortars has demonstrated that the hot-mixing technique involved the direct addition of quicklime (CaO) to the aggregates and water during mortar preparation. This process promotes the formation of lime clasts and may locally preserve incompletely hydrated CaO or its transformation products. As reported by Masic et al. [52], these lime clasts should not be regarded as manufacturing defects; rather, they are an intentional feature of Roman construction technology and play an important role in enhancing the self-healing capacity of the mortar (Figure 26, Table 9).

**Table 9:** Qualitative phase analysis by X-Ray diffraction of the plaster mortar.

Phase Name	Formula	Figure of Merit	Phase Reg. Detail	DB Card Number
O_2_Si	O_2_Si	0.628	User (COD)	2300370
Calcium Carbonate	CaCO_3_	0.894	User (COD)	7022027
CaO_2_	CaO_2_	1.724	User (COD)	1530292
Bazhenovite	Ca2 H9.2 O8 S2.19	3.371	User (COD)	9003813
Enstatite	Ca0.07 Mg1.93 O6 Si2	1.984	User (COD)	9004029
Albite	Al K0.2 Na0.8 O8 Si3	1.525	User (COD)	9000681
Vesuvianite	Ca10 (Mg Fe) Al4 (Si O4)_5_ (Si2 O7)_2_ (OH)_4_	1.391	User (COD)	1011221

The presence in the mortar structure of the phases calcite (CaCO_3_), silica (SiO_2_) from sand, silicates, and aluminosilicates confirms the contribution of reactions between calcium hydroxide and silica or alumina in forming mineral compounds that confer durability to Roman mortar, according to the theory developed by Marie Jackson.

## 4. Conclusions

The characterization of the mortar from the eastern wall of the Sucidava Fortress included analyses of its macroscopic and microscopic structure, mineralogy, morphology, elemental chemical composition, and phase composition.

The materials were characterized whose structure and composition confirm their status as mortar: a masonry mortar with larger granulation, mortar type *Opus caementicium* [62,63], which also has a protective plaster layer, and a fine plaster mortar containing brick, mortar type Opus signinum [64,65,66].

The phase composition of the mortars shows the presence of siliceous aggregates, formed from C-S-H and C-A-S-H gels, resulting from mineral transformations in the mortar—phases that strengthen the mortar structure and confer durability [8,10,12,13,14,64,67].

Both identified mortars contain lime particles and bone-derived particles. The bone particles, likely originating from burned bone ash used as a local substitute for unavailable pozzolanic ash, introduce phosphorus into the mortar.

According to recent findings, the presence of phosphorus through hydroxyapatite and the formation of Ca–P–Si compounds as a result of using bone ash positively influence the properties of hydraulic mortars: faster setting, higher initial strength, compressive and freeze–thaw resistance, dense microstructure with stronger bonds, improved intergranular adhesion, and better resistance to moisture and degradation [53,54,55,56,57,58,59,60,61].

From a historical perspective, the results demonstrate that, despite the political and administrative decline of the Roman Empire, high-quality mortar technology persisted in the Eastern Roman (Byzantine) regions. Chronology and the characterizations indicate that the mortars studied are similar to those from Asia Minor and the Balkans, reflecting continuity in construction practices and technological knowledge [67,68,69,70,71,72].

From a technical and environmental standpoint, the characteristics of Roman mortar include enhanced durability and the use of locally available materials, making it a sustainable construction solution in ancient times.

## Figures and Tables

**Table 1:** Characterized samples from the east wall of the Sucidava Fortress.

No.	Mortar Type	Analysis	Sampling Location	Sample No.
1	Masonry mortar	Macroscopic Features	Eastern outer wall	1 M
		Cathodoluminescence		1 CL-G
		Granulometric analysis		
		Optical Microscopy		1 MO
		SEM EDS		1 SEM EDS
		XRD		1 XRD
2	Masonry mortar with lime finishing coat	Macroscopic Features	Eastern outer wall	2 M
		Optical Microscopy		2 MO
		SEM EDS		2 SEM-EDS
		XRD		2 XRD
3	Masonry mortar with finishing plaster	Macroscopic Features	Inner east wall	3 M
		Optical Microscopy		3MO
		SEM EDS		3 SEM-EDS
		XRD		3 XRD
4	Plaster mortar	Macroscopic Features	Inner east wall	4 M
		Optical Microscopy		4 MO
		SEM ED		4 SEM EDS

**Table 5:** Chemical composition determined by SEM-EDS obtained by 3 point-scans presented in Figure 19.

Spectrum Label (wt%)	Spectrum 1	Spectrum 2	Spectrum 3
O	45.63	40.24	53.33
Na	0.23	-	0.23
Mg	0.64	0.53	1.92
Al	1.71	1.78	2.66
Si	4.61	4.88	6.79
P	15.75	17.42	0.34
S	-	-	0.15
K	0.49	0.60	0.62
Ca	29.91	33.25	31.07
Mn	-	-	0.46
Fe	1.03	1.29	2.42
Total	100.00	100.00	100.00

## Data Availability

The original contributions presented in this study are included in the article. Further inquiries can be directed to the corresponding author.
